# Effectiveness and Acceptability of Asynchronous Digital Health in Asthma Care: Mixed Methods Systematic Review

**DOI:** 10.2196/57708

**Published:** 2024-12-03

**Authors:** Nazim Uzzaman, Victoria Hammersley, Kirstie McClatchey, Jessica Sheringham, Diksha Singh, GM Monsur Habib, Hilary Pinnock

**Affiliations:** 1 Usher Institute The University of Edinburgh Edinburgh United Kingdom; 2 Directorate of Public Health NHS Tayside Dundee United Kingdom; 3 School of Medicine University of Dundee Dundee United Kingdom; 4 Institute of Epidemiology and Health Care University College London London United Kingdom; 5 Community Respiratory Centre Bangladesh Primary Care Respiratory Society (BPCRS) Khulna Bangladesh

**Keywords:** digital health, asthma, asynchronous, asthma care, effectiveness, acceptability, mixed-methods review, systematic review, barrier, remote synchronous, chronic respiratory disease, self-management, digital technology, asynchronous consultation, caregiver, PRISMA

## Abstract

**Background:**

Asynchronous digital health (eg, web-based portal, text, and email communication) can overcome practical barriers associated with in-person and remote synchronous (real-time) consultations. However, little is known about the effectiveness and acceptability of asynchronous digital health to support care for individuals with asthma (eg, asthma reviews).

**Objective:**

We aimed to systematically review the qualitative and quantitative evidence on the role of asynchronous digital health for asthma care.

**Methods:**

Following Cochrane methodology, we searched 6 databases (January 2001-July 2022; search update: September 2023) for quantitative, qualitative, or mixed methods studies supporting asthma care using asynchronous digital health. Screening and data extraction were duplicated. We assessed the risk of bias in the clinical outcomes of randomized controlled trials included in the meta-analysis using the revised Cochrane risk of bias tool. For the remaining studies, we evaluated the methodological quality using the Downs and Black checklist, critical appraisal skills program, and mixed methods appraisal tool for quantitative, qualitative, and mixed methods studies, respectively. We determined the confidence in the evidence using the GRADE (Grading of Recommendations, Assessment, Development, and Evaluation) criteria. We conducted a meta-analysis of trial data and a thematic analysis of qualitative data.

**Results:**

We included 30 studies (20 quantitative, 6 qualitative, and 4 mixed methods) conducted in 9 countries involving individuals with asthma, their caregivers, and health care professionals. Asynchronous digital consultations linked with other functionalities, compared to usual care, improved asthma control (standardized mean difference 0.32, 95% CI 0.02-0.63; *P*=.04) and reduced hospitalizations (risk ratio 0.36; 95% CI 0.14-0.94; *P*=.04). However, there were no significant differences in quality of life (standardized mean difference 0.16; 95% CI –0.12 to 0.43; *P*=.26) or emergency department visits (risk ratio 0.83; 95% CI 0.33-2.09; *P*=.69). Patients appreciated the convenience of asynchronous digital health, though health care professionals expressed concerns. Successful implementation necessitated an organizational approach. Integrative synthesis underscored the ease of asking questions, monitoring logs, and medication reminders as key digital functionalities.

**Conclusions:**

Despite low confidence in evidence, asynchronous consultation supported by digital functionalities is an effective and convenient option for nonemergency asthma care. This type of consultation, well accepted by individuals with asthma and their caregivers, offers opportunities for those facing challenges with traditional synchronous consultations due to lifestyle or geographic constraints. However, efficient organizational strategies are needed to manage the associated workload.

**Trial Registration:**

PROSPERO CRD42022344224; https://www.crd.york.ac.uk/prospero/display_record.php?RecordID=344224

**International Registered Report Identifier (IRRID):**

RR2-10.1371/journal.pone.0281538

## Introduction

Asthma, with an estimated 262 million cases worldwide, is the most prevalent chronic respiratory disease [1]. While some countries have witnessed a decline in asthma-related hospitalizations and deaths [1], asthma still poses an unacceptable burden on health care systems and society at large, disrupting both work and family life [2]. An asthma review helps assess asthma control, adjust management strategies, support self-management education [2,3], and understand patients' thoughts and concerns [4]. While well-controlled individuals should be reviewed at least once a year [3], those with poor asthma control or newly diagnosed cases will require more frequent review. However, challenges such as poor attendance at asthma clinics, time constraints, limited resources, and competing agendas during consultations hinder effective reviews [5]. Given the increasing access to the internet and mobile technology [6,7], digital health emerges as an innovative approach that could improve health outcomes [8] while reducing avoidable clinic visits [9].

Digital health interventions can be delivered either synchronously (real-time interaction) or asynchronously (no real-time interaction, for example, sharing clinical information from patients through email that allows a health care professional to review the data and provide feedback later), potentially offering convenient and accessible health care [10]. Synchronous remote asthma reviews conducted via telephone have been in use for about 2 decades [11], receiving widespread acceptance, while videoconferencing experienced a substantial increase, especially during the COVID-19 pandemic [12,13]. Although asynchronous digital health can overcome the time constraints linked to in-person and synchronous remote reviews and has the potential to support care of the large number of people with asthma, it remains underresearched [14].

Existing systematic reviews have synthesized evidence on remote asthma monitoring, medication adherence, and self-management support using a broad range of digital technologies [15-20]. However, none of these reviews focused on the role of 2-way asynchronous communication between patients and health care professionals. Specifically, they have not synthesized the evidence of the effectiveness of asynchronous digital health (either as an isolated intervention or in combination with other modalities) nor explored patients' and professional stakeholders' perspectives on asynchronous consulting. This is timely given the increasing use of questionnaires promoted to support primary care practices reviewing patients with long-term conditions [21,22]. We, therefore, aimed to synthesize the quantitative and qualitative evidence to derive recommendations for policy and practice on the use of asynchronous digital health for asthma care. Our objectives were to (1) assess the effects of interventions using asynchronous digital health on clinical outcomes (ie, asthma control, quality of life, emergency department visits, and hospitalizations) compared to usual care; (2) describe the digital functionalities used for asynchronous consulting; (3) explore the views and experience of patients and health care professionals on asynchronous digital health; and (4) integrate the quantitative and qualitative synthesis to derive implications for clinical practice and policymaking.

## Methods

### Overview

We conducted a mixed methods systematic review following results-based convergent design [23]. We adhered to Cochrane methodology [24] and used the PRISMA (Preferred Reporting Items for Systematic Reviews and Meta-Analyses) guidelines to report our review findings [25]. The review is registered at PROSPERO (CRD42022344224), and the protocol is published [26].

### Search Strategy and Selection Criteria

We developed a search strategy involving the review authors and a senior librarian from the University of Edinburgh. We searched MEDLINE, Embase, Scopus, PsycInfo, CINAHL, and Cochrane Library (S1 in Multimedia Appendix 1) from 2001 (when internet accessibility surged with third-generation cellular technologies) [27] to July 2022. We updated the search in September 2023. We placed no language restrictions on the database search and kept the option to translate relevant quantitative studies to English [28]. However, we only considered qualitative and mixed-methods studies written in English due to the potential loss of nuance in translation [29].

After the search, all identified records were saved in EndNote 20 (Clarivate Analytics), with duplicates removed using SRA Deduplicator software [30]. In total, 2 authors (NU and MH) independently screened titles, abstracts, and full-text papers for eligibility against the criteria (S2 in Multimedia Appendix 1), using Covidence [31]. Any discrepancies were settled through team discussion (HP, VH, KM, and JS). The search results are presented in a PRISMA flow diagram.

### Quantitative Outcome Measurements

Our primary (clinical) outcomes from quantitative data were asthma control or quality of life measured with any validated tools and acute attacks (eg, emergency department visits, hospitalizations, or unscheduled care) [32-34]. We were interested in between-group differences at the first follow-up assessment postintervention. Where multiple assessment tools for an outcome were reported, we used the most frequently reported validated measure (eg, asthma control test [ACT] [32], childhood-asthma control test [C-ACT] [35]) in the meta-analysis.

### Qualitative Phenomena of Interest

We included studies that explored the views and experiences of patients and professional stakeholders on asynchronous digital health (with or without other modes of communication) for asthma care.

### Data Extraction and Management

NU and MH independently extracted quantitative data, and NU and DS independently extracted qualitative data from the included studies. HP checked the accuracy of data across text, tables, and meta-analyses.

### Data Analysis Addressing Our Review Objectives

Textbox 1 summarizes the methods of data analysis used to address our review objectives.

**Data analysis for our 4 objectives.**

- Effectiveness of asynchronous digital health. Clinical outcomes (asthma control, quality of life, emergency department visits, and hospitalizations) from eligible randomized control trials were pooled in the meta-analysis. Cochrane’s Review Manager (RevMan 2020, version 5.4.1) was used for conducting the meta-analysis. The sample size of each study in the meta-analysis was based on the reported number of participants in the analysis as provided by the study authors. For asthma control and quality of life, standardized mean difference (for different scale metrics) with 95% CI was calculated using the inverse variance method. For emergency department visits and hospitalizations, we calculated risk ratio (RR) with 95% CI using the Mantel-Haenszel method. A *P* value less than .05 was considered statistically significant for the overall effect.
- Digital functionalities. We identified the digital functionalities used in the included quantitative and mixed methods studies and illustrated these graphically.
- Views and experiences. We used thematic synthesis to combine the findings of studies that described the views and experiences of patients and health care professionals on asynchronous digital health for asthma care, following recognized methodology [36] (see S3 in Multimedia Appendix 1).
- Integrative synthesis. We integrated the quantitative and qualitative findings following the Cochrane Handbook and presented them in a matrix [24,37], illustrating findings from the qualitative synthesis (ie, preferred digital functionalities, and implementation facilitators) aligned with intervention effects on the clinical outcomes.

### Heterogeneity and Reporting Bias

We assessed statistical heterogeneity through visual inspection of forest plots, chi-square, and *I*^2^ tests for our clinical outcome [38]. We used a random-effects model to account for heterogeneity in the study intervention, population, and settings. The limited number of studies (<10) in the meta-analysis precluded creation of a funnel plot to assess publication bias [39].

### Subgroups and Sensitivity Analysis

Our *a priori* planned subgroups were age (child or adult), high- or low-income countries, asthma severity, and intensity of intervention, and we planned to include an additional subgroup if that were suggested by the qualitative synthesis. We undertook a sensitivity analysis of our meta-analysis comparing the intervention with usual care, excluding studies at high risk of bias and those that could potentially influence results due to study design or inadequacies in data reporting.

### Dealing With Missing Data

We contacted the author(s) of included studies to collect any incomplete or missing data but did not perform any statistical calculation to impute missing data into the meta-analysis.

### Methodological Quality Assessment

We used the revised Cochrane risk-of-bias tool [40] and assessed risk of bias for clinical outcomes within the randomized control trials (RCTs) that were included in the meta-analysis. We assessed the methodological quality of the remaining quantitative studies with diverse study designs using the Downs and Black checklist [41-43]. We used the critical appraisal skills program for qualitative studies [44,45] and mixed methods appraisal tool for mixed methods studies to appraise their methodological quality [46].

### Assessment of Confidence in Evidence

Using Cochrane’s GRADEpro GDT software [47], we assessed confidence in evidence for the clinical outcomes following the 5 GRADE (Grading of Recommendations, Assessment, Development, and Evaluations) criteria (risk of bias, consistency of effect, imprecision, indirectness, and publication bias) [24]. We used the Grades of Recommendation, Assessment, Development, and Evaluation-Confidence in the Evidence from Qualitative Reviews (CERQual) approach to assess confidence in synthesized qualitative findings using the interactive summary of qualitative findings tool [48,49].

## Results

### Study Selection

**Figure 1:**
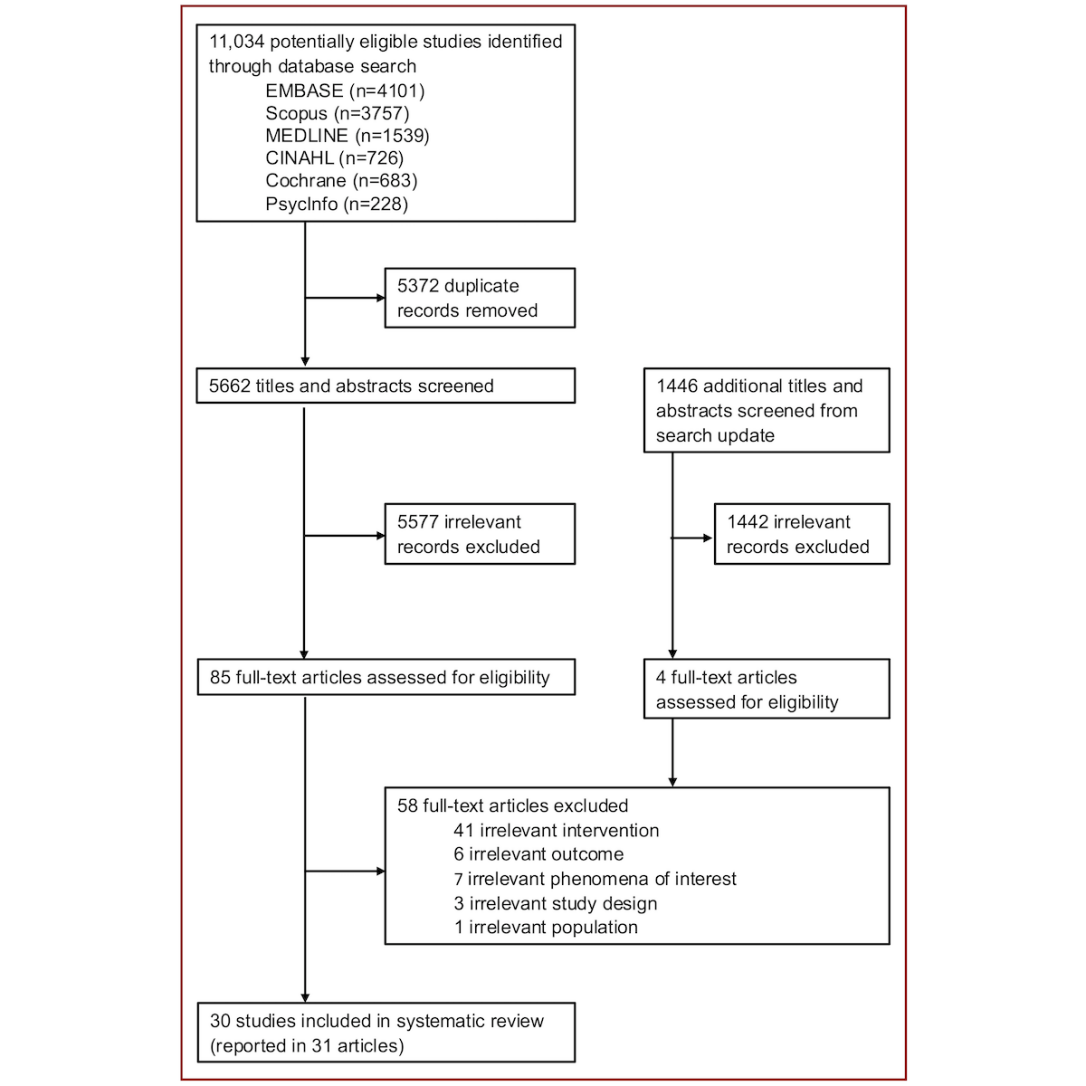
Study selection flow.

We identified 11,034 records from 6 databases (Figure 1). After deduplication, 5662 titles and abstracts were screened, leading to the assessment of 85 full-text articles. We included 30 studies (31 unique papers) after combining 2 reports from the same study [50-80]. Out of the 1446 records screened in the search update, 4 full-text articles were assessed, but none met the eligibility criteria for inclusion in the review. The list of excluded articles and reasons is detailed in S4 in Multimedia Appendix 1.

### Characteristics of Included Studies

Of the 30 included studies, 20 were quantitative [50,51,53-57,60,61,64,65,67-69,72-74,76,78-80], 6 were qualitative [52,58,66,70,71,77], and 4 were mixed-methods studies [59,62,63,75]. These studies, involving individuals with asthma, their caregivers, and health care professionals, were conducted in 9 countries, predominantly from the United States (n=14) [50,52,53,55,56,58-62,66,69,70,79] and the Netherlands (n=9) [65,71-78]. The quantitative studies encompassed various designs, including RCTs (n=14) [51,53,55,57,60,61, 65,67,68,72,74,78-80]. Of the RCTs, a cluster trial was reported in 2 papers [64,65], detailing the main trial outcomes in 1 paper [65], and the process evaluation in the other [64]. The summary of included studies is presented in S5 and S6 in Multimedia Appendix 1. The pool of studies varies for the different objectives, so further characteristics are described by objective in Table 1.

**Table 1:** Objective-wise evidence summary.

Variables				Values
**Studies addressing objective 1: effectiveness of asynchronous digital health**				16 studies [51,53,55,57,60,61,65,67-69,72-74,76,78,79]; 2328 participants (1217 children, 1111 adults)
**Outcomes included in the meta-analysis**				
		Asthma control	7 studies [57,60,61,65,72,74,78]	
		Quality of life	5 studies [51,55,65,74,78]	
		Emergency department visits	5 studies [55,57,60,68,72]	
		Hospitalizations	4 studies [55,60,67,72]	
**Outcomes included in narrative synthesis**				
		Asthma control	5 studies [51,53,69,73,76]	
		Quality of life	5 studies [53,60,68,73,76]	
		Emergency department visits	4 studies [51,53,69,79]	
**Studies addressing objective 2: digital health functionalities used**				21 [50,51,53-57,59-61,65,67-69,72-74,76,78-80]; 2793 participants (1614 children, 1179 adults)
**Quantitative studies**				
		RCT^a^	14 studies [51,53,55,57,60,61,65,67,68,72,74,78-80]	
		Pre-post	2 studies [56,69]	
		Quasi-experimental	1 study [73]	
		Observational comparative	1 study [54]	
		Quantitative survey	1 study [50]	
		Long-term follow-up of an RCT	1 study [76]	
**Mixed-methods study**				1 study [59]
**Studies addressing objective 3: views and experiences**				10 studies [52,58,59,62,63,65,70,71,75,77]; 450 participants (173 parents, 151 patients, 87 physicians, 35 nurses, and 4 pharmacists)
**Qualitative studies**				6 studies [52,58,66,70,71,77]
	Interviews (individual or group or dyadic)			3 studies [52,58,66]
	Interviews and focus groups			2 studies [70,77]
	Qualitative survey			1 study [71]
**Mixed methods studies**				4 studies [59,62,63,75]
	Sequential exploratory			2 studies [63,75]
	Implementation study			1 study [59]
	Mixed-methods survey			1 study [62]
**Studies addressing objective 4: integrative synthesis**				17 studies [51,52,55,57-61,63,65-68,71,72,74,78]; 1886 participants (1576 from quantitative, 310 from qualitative studies)
	Quantitative evidence (RCTs in meta-analysis)			11 studies [51,55,57,60,61,65,67,68,72,74,78]
	Qualitative evidence			6 studies [52,58,59,63,66,71]

^a^RCT: randomized controlled trial.

### Effectiveness of Asynchronous Digital Health (Objective 1)

#### Overview

**Figure 2:**
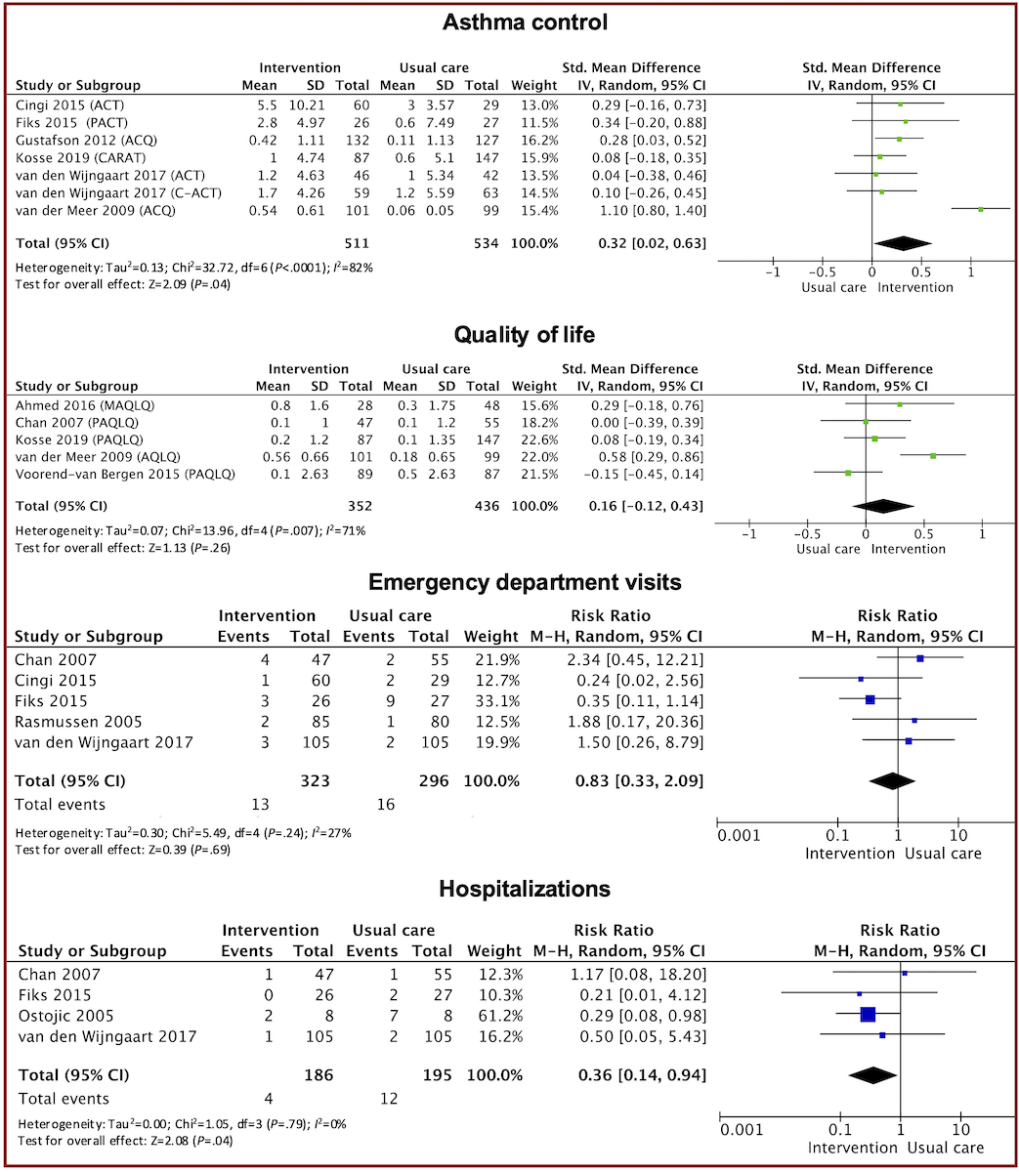
Forest plot of studies comparing the intervention with usual care on asthma control [57,60,61,65,72,74], quality of life [51,55,65,74,78], emergency department visits [55,57,60,68,72], and hospitalizations [55,60,67,72].

No studies reported asynchronous digital consultations as an isolated intervention. Therefore, we assessed the effectiveness of asynchronous digital consultations supported by other digital health functionalities on the clinical outcomes compared to usual care. The meta-analyses are in Figure 2 [51,55,57,60,61,65,67,68,72,74,78], and the sensitivity analysis is in S7 in Multimedia Appendix 1. The risk of bias for individual clinical outcomes is illustrated in a figure in S8 in Multimedia Appendix 1. Quantitative studies excluded from the meta-analysis due to heterogeneity in study design or data reporting are detailed in S9 in Multimedia Appendix 1 through narrative synthesis.

#### Asthma Control

We were able to retrieve data from 7 trials for meta-analysis comparing asthma control between intervention and usual care groups [57,60,61,65,72,74,78]. In total, 2 trials used both ACT and C-ACT [72,78], 1 used only ACT [57], and the others used asthma control questionnaire (ACQ) [61,74], pediatric asthma control tool [60], and control of allergic rhinitis and asthma test [65].. Of these 7 trials, we excluded a study from the meta-analysis as it reported undifferentiated ACT and C-ACT scores (which are not interchangeable) but included it in the sensitivity analysis [78]. The pooled meta-analysis revealed a statistically significant improvement in asthma control favoring the intervention group (standardized mean difference (SMD) 0.32; 95% CI 0.02-0.63; *P*=.04).

A sensitivity analysis, excluding the study at high risk of bias [57], the cluster RCT [65], and the study that induced substantial heterogeneity (*I*^2^>50%) [74], did not change the conclusion for asthma control (SMD 0.20, 95% CI 0.03-0.37; *I*^2^=0%; *P*=.02). However, the *P* value edged just beyond the point of statistical significance (SMD 0.14, 95% CI −0.01 to 0.29; *I*^2^=0%; *P*=.06) when we included the study that reported undifferentiated ACT and C-ACT scores for asthma control [78].

#### Quality of Life

Data from 5 trials were obtained for the meta-analysis [51,55,65,74,78]. Among these, 3 trials used pediatric asthma quality of life questionnaire (PAQLQ) [55,65,78], while the others used mini asthma quality of life questionnaire (MAQLQ) [51] and asthma quality of life questionnaire (AQLQ) [74]. The pooled estimate indicated no statistically significant difference in quality of life when comparing intervention with usual care (SMD 0.16; 95% CI –0.12 to 0.43; *P*=.26). We could not perform sensitivity analysis for quality of life due to a lack of studies following the exclusion of those that could potentially bias results [51,55,65,74].

#### Emergency Department Visits

In total, 5 trials were incorporated into the meta-analysis for emergency department visits [55,57,60,68,72]. The pooled estimate revealed no statistically significant difference in emergency department visits between intervention and usual care (RR 0.83; 95% CI 0.33-2.09; *P*=.69). The sensitivity analysis did not change the conclusion (RR 1.01; 95% CI 0.37-2.77; *I*^2^=33%; *P*=.99).

#### Hospitalizations

In total, 4 trials were included in the meta-analysis for hospitalization [55,60,67,72]. The pooled estimate showed that the intervention group had a statistically significant reduced risk of hospitalization compared to the usual care group (RR 0.36; 95% CI 0.14-0.94; *P*=.04). However, the statistical significance of the risk of hospitalization became inconclusive (RR 0.52; 95% CI 0.11-2.42; *I*^2^=0%; *P*=.40) after the sensitivity analysis.

### Digital Health Functionalities Used (Objective 2)

**Figure 3:**
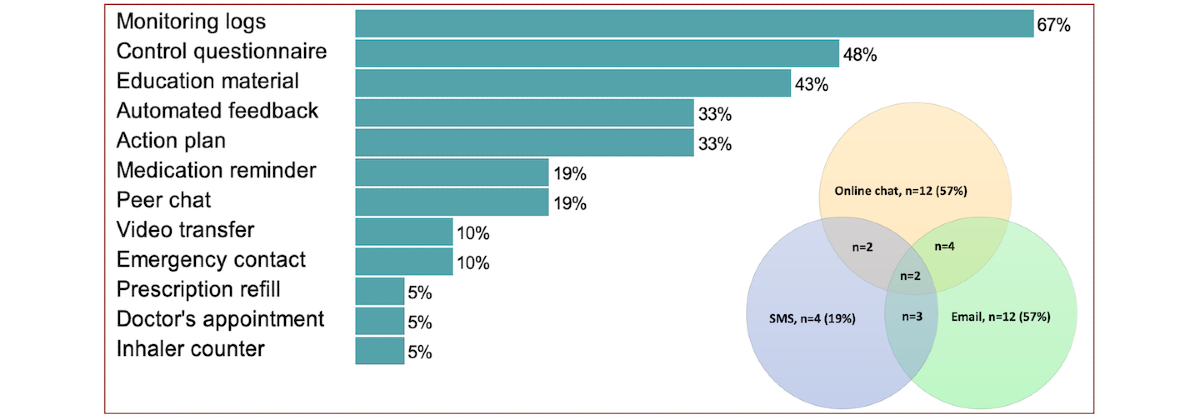
Digital functionalities reported in 21 studies [50,51,53-57,59-61,65,67-69,72-74,76,78-80].

In total, 21 studies documented the use of digital functionalities, with all of them incorporating either online chat (n=12) [50,53,54,57,59,60,65,72-74,76,79] and email (n=12) [51,55,56,59-61,68,69,74,76,78,80], and SMS (n=4) [67,69,74,76] for asynchronous communication between patients or their caregivers and health care professionals. The response time ranged from within 24 hours [51,54] to 48 hours [61,72] and 72 hours [78]. No studies explicitly addressed the role of non-digital support in using digital functionalities. However, in an RCT where both groups used a web portal, similar usage was observed, despite the intervention group receiving additional support through home visits by community health workers [53]. Figure 3 [50,51,53-57,59-61,65,67-69,72-74,76,78-80] illustrates the digital health functionalities used in the included studies, with additional details available in S10 in Multimedia Appendix 1.

### Views and Experiences of Patients and Health Care Professionals (Objective 3)

The thematic synthesis yielded four themes: (1) acceptability of routine asthma reviews, (2) advantages and disadvantages of asynchronous digital health, (3) implementation barriers and facilitators, and (4) preferred digital functionalities.

#### Theme 1: Acceptability of Routine Asthma Reviews

In total, 3 studies explored the views of patients or their carers and health care professionals on routine asthma reviews [63,66,75]. Most adolescents with well-controlled asthma perceived in-person routine reviews as unnecessary and preferred seeking medical help only when their symptoms worsened [75]. A few individuals with poorly controlled asthma were bothered by regular face-to-face medical reviews, mentioning that they learned to live with their symptoms. Both groups were enthusiastic about internet-based reviews and found email communication and electronic consultation useful [75].

Most patients or caregivers found asynchronous digital health feasible and accessible [63,66,75]; however, none wanted to completely replace traditional face-to-face consultations [66]. Health care professionals echoed convenience but noted the clinical limitations of remote reviews [63].

#### Theme 2: Advantages and Disadvantages of Asynchronous Digital Health

In total, 6 studies reported the benefits of asynchronous digital health [52,62,66,70,71,75]. Most parents of children with asthma found asynchronous digital health to be a convenient method for reviewing and managing their child's asthma while managing work and family responsibilities [52,66].

Parents felt that asynchronous digital health improved day-to-day care management and reduced unplanned provider visits. They expressed a sense of reassurance in being able to monitor their child's condition, receive remote guidance from health care professionals, and support self-management [66].

Health care professionals viewed text messaging and emails as effective means to connect with patients, enhancing communication, and fostering compliance and treatment adherence [62,70].

In total, 4 studies reported limitations of asynchronous digital health [59,66,70,71]. Health care professionals perceived that asynchronous digital health was not suitable for patients with poor perception of their asthma symptoms or needing emergency care [70]. They also expressed concerns that the lack of physical examination and reduced face-to-face contact associated with this approach could negatively affect clinical decision-making [71]. Some parents and children with asthma found frequent monitoring data sharing to be burdensome [59,66].

#### Theme 3: Implementation Barriers and Facilitators

In total, 6 studies reported the barriers to implementing asynchronous digital health in routine practice [59,66,70,71,75,77]. The major barriers, as perceived by most health care professionals, included lack of integration with electronic health records (EHRs) so that data have to be transferred manually [59,71,77], poorly coordinated workflow [59,71,77], high workload [70,71,77], and the absence of financial reimbursement [59,70,77].

Some health care professionals also mentioned that a lack of internet access (eg, because of rurality) and the unavailability of electronic devices or the lack of skills using them by some patients acted as barriers [59,71,77]. Other barriers include data security [70,71,77], negative attitudes (not convinced of the added value of digital health in daily practice) [71,75,77], missing interpersonal relationships [71,77] and a lack of training for practices [59].

In total, 4 studies reported implementation facilitators [52,59,66,71]. Both health care professionals and parents of children with asthma recognized that accessible “easy” 2-way communication, prompt responsiveness from practices and families, as well as a positive attitude and commitment towards adopting asynchronous digital health were facilitators [52,59,71].

Health care professionals also emphasized the importance of efficient task allocation among the practice staff in facilitating successful implementation [59,71].

Other facilitators included user-friendliness of the digital system [66,71], shared decision-making, positive attitude or commitment, and freeing up time for complex patients [71].

#### Theme 4: Preferred Digital Functionalities

In total, 5 studies reported preferred digital functionalities [52,58,63,66,71]. Most patients wanted the flexibility to ask quick questions [63], receive tailored information about asthma [63,66,71], log and visualize the trend of their peak flows, symptom scores, and medication usage [63], and receive medication reminders [52,58]. Health care professionals acknowledged the importance of a flexible approach to patient reviews but expressed concerns about available resources [63]. They also thought that logs (especially symptoms, but also peak flows) could engage patients in “thoughtfully” reporting their asthma status and would inform the assessment of control and management strategies.

In contrast to the priority attached by professionals to logging, most patients acknowledged that in reality, they checked their peak flow “rarely” or only when their asthma was getting worse [63]. The reasons for not measuring every day were varied. Some forgot, while others felt uncomfortable doing it in front of people, but many suggested it was unnecessary as they knew their asthma and could assess status by how they felt.

Most parents reported checking their emails “frequently,” but this ranged from once a week to several times a day. Some participants preferred to be contacted by SMS text message or smartphone application. Others preferred a phone call or stated they were “okay” with any method of contact (text, application, email, and call) [52]. Some participants highlighted that too frequent messaging was counterproductive [58].

### Integration of Qualitative and Quantitative Synthesis (Objective 4)

Implications from qualitative synthesis (preferred digital functionalities and implementation facilitators) were juxtaposed with trial findings for clinical outcomes and integrated to see if those implications had a role in improving clinical outcomes (Table 2). Out of the 6 RCTs [57,60,61,65,72,74] that showed positive improvement in asthma control, all had monitoring logs [57,60,61,65,72,74], 5 had ease of asking quick questions [57,60,65,72,74], 5 provided tailored asthma information [60,61,65,72,74], 3 had medication reminders [57,61,65], 3 had digital action plans [60,61,72], 1 was linked with the EHR, and 4 had an organized workflow with a specific person designated to respond to patients’ queries [61,65,72,74]. One study in which asthma control deteriorated had monitoring logs only [78]. Three RCTs that reported improvement in quality of life, all had ease of asking quick questions, monitoring logs, tailored asthma information, and organized workflow [51,65,74]. One RCT that reported no change in quality of life also had these features [55]. One study, where quality of life deteriorated, had monitoring logs only [78]. Of 2 of the 5 RCTs [55,57,60,68,72] that reported reduced risk of emergency department visits, 1 had medication reminders [57], and the other was linked with the EHR [60]. Of the 3 RCTs [55,68,72] that reported increased risk of emergency department visits, none had medication reminders nor were linked with EHR. However, 2 out of these 3 RCTs had ease of asking quick questions, monitoring logs, tailored asthma information, and organized workflow [55,72]. There was no specific pattern to differentiate the trials that reported increased risk of hospitalization [55] compared with those that reported reduced risk of hospitalization [60,67,72]. Overall, the ease of asking quick questions, medication reminders, tailored asthma information, and organized workflow emerged as important factors that might positively affect the intervention outcomes.

**Table 2:** Integrative synthesis.

Study	Implications from qualitative synthesis							Trial outcomes from meta-analyses			
	Preferred functionalities					Facilitators		Asthma control	HRQoL^a^	ED visits^b^	Admissions
	Quick Q^c^	Logs^d^	Reminder^e^	PAAP^f^	Info^g^	EHR link^h^	Responder^i^	Measure: MD (95% CI)^j^	Measure: MD (95% CI)	RR (95% CI)^k^	RR (95% CI)
Ahmed et al (2016) [51]	✓	✓	x	✓	✓	✓	Nurse	—	MAQLQ^l^: 0.50 (–0.27 to 1.27)	—	—
Chan et al (2007) [55]	✓	✓	x	x	✓	x	Nurse	—	PAQLQ^m^: 0.00 (–0.43 to 0.43)	2.34 (0.45 to 12.21)	1.17 (0.08 to 18.20)
Cingi et al (2015) [57]	✓	✓	✓	x	x	x	NR^n^	ACT^o^: 2.50 (–0.39 to 5.39)	—	0.24 (0.02 to 2.56)	—
Fiks et al (2015) [60]	✓	✓	x	✓	✓	✓	NR	PACT^p^: 2.20 (–1.21 to 5.61)	—	0.35 (0.11 to 1.14)	0.21 (0.01 to 4.12)
Gustafson et al (2012) [61]	x	✓	✓	✓	✓	x	Study team	ACQ^q^: –0.31 (–0.58 to –0.04)	—	—	—
Kosse et al (2019) [65]	✓	✓	✓	x	✓	x	Pharmacist	CARAT^r^: 0.40 (–0.89 to 1.69)	PAQLQ: 0.10 (–0.23 to 0.43)	—	—
Ostojic et al (2005) [67]	x	✓	✓	x	✓	x	Asthma specialist	—	—	—	0.29 (0.08 to 0.98)
Rasmussen et al (2005) [68]	x	✓	x	✓	x	x	NR	—	—	1.88 (0.17 to 20.36)	—
van den Wijngaart et al (2017) [72]	✓	✓	x	✓	✓	x	Asthma team	ACT: 0.20 (–1.90 to 2.30); C–ACT^s^: 0.50 (–1.26 to 2.26)	—	1.50 (0.26 to 8.79)	0.50 (0.05 to 5.43)
van der Meer et al (2009) [74]	✓	✓	x	x	✓	x	Asthma nurse	ACQ: –0.48 (–0.60 to –0.36)	AQLQ^t^: 0.38 (0.20 to 0.56)	—	—
Voorend-van Bergen et al (2015) [78]	x	✓	x	x	x	x	Nurse	ACT or C–ACT: –0.20 (–1.72 to 1.32)	PAQLQ: –0.40 (–1.18 to 0.38)	—	—

^a^HRQoL: health-related quality of Life.

^b^ED visits: emergency department visits.

^c^Quick Q: ease of asking quick questions.

^d^Logs: monitoring logs.

^e^Reminder: medication reminder.

^f^PAAP: personalized asthma action plan.

^g^Info: tailored asthma information.

^h^EHR link: linked with electronic health record.

^i^Responder: assigned responder to patient queries.

^j^MD (95% CI): mean difference (95% CI).

^k^RR (95% CI): risk ratio (95% CI).

^l^MAQLQ: mini asthma quality of life questionnaire.

^m^PAQLQ: pediatric asthma quality of life questionnaire.

^n^NR: not reported.

^o^ACT: asthma control test.

^p^PACT: pediatric asthma control tool.

^q^ACQ: asthma control questionnaire.

^r^CARAT: control of allergic rhinitis and asthma test.

^s^C-ACT: childhood asthma control test.

^t^AQLQ: asthma quality of life questionnaire.

### Methodological Quality

The Downs and Black scores ranged from 6 to 24 for the other quantitative studies that were not included in the meta-analysis. Methodological quality was good in 3 studies (range: 20-25) [53,79,80], fair (range: 15-19) in 2 studies [69,76] and poor (≤14) in the other 4 studies [50,54,56,73]. Among qualitative studies, 1 had high quality [77], while 5 raised some concerns [52,58,66,70,71]. Of the 4 mixed-method studies, 1 demonstrated high quality [63], and 3 raised some concerns [59,62,75]. Methodological quality assessments for qualitative and mixed-methods studies are detailed in S11 and S12 in Multimedia Appendix 1.

### Confidence in Evidence

Using the GRADE approach, the overall certainty of evidence for the clinical outcomes was judged as very low for asthma control and low for quality of life, emergency department visits, and hospitalization. Downgrading of the certainty was mostly due to the risk of bias and imprecision (S13 in Multimedia Appendix 1). The GRADE-CERQual assessment of confidence revealed that summary review findings of the qualitative studies ranged from low to high quality. Perspectives of routine reviews, advantages and disadvantages of using asynchronous digital health, implementation facilitators, and preferred digital functionalities had moderate confidence. Limitations of using asynchronous digital health revealed low confidence, and implementation barriers revealed high confidence (S14 in Multimedia Appendix 1).

## Discussion

### Summary of Findings

Our systematic review identified 30 studies involving people with asthma (and their caregivers) and health care professionals from 9 countries. Overall, we identified a statistically significant improvement in asthma control and a reduced risk of hospitalization when comparing interventions using asynchronous digital health with usual care. However, no statistically significant differences were observed in quality of life and emergency department visits. Certainty of evidence was very low for asthma control and low for quality of life, emergency department visits, and hospitalizations. Patients liked the convenience of asynchronous digital health, while health care professionals had some reservations. Effective implementation requires an organizational approach. Integrative synthesis highlighted the importance of functionalities such as ease of asking quick questions, monitoring logs, and medication reminders.

### Strength and Limitations

A strength of this review is its inclusive design, encompassing various study types and integrating quantitative and qualitative findings. We conducted a comprehensive database search, aided by a senior librarian, and the meta-analysis was reviewed by a senior statistician from the University of Edinburgh. Heterogeneity was a concern for some clinical outcomes in the meta-analysis, but our sensitivity analysis increased the reliability of the pooled estimates. We maintained an openness to non-English papers in quantitative studies and included 1 in Russian [54]. We did not deviate from the methodology outlined in the published protocol [26]. While our GRADE assessment reflected low to very low confidence in clinical outcomes, this was influenced by self-reported outcome measures and diverse tools for asthma control and quality of life. This underscores the need for standardized outcomes in trials [81]. In total, 2 independent reviewers screened titles, abstracts, and full texts, resolving discrepancies through discussion with other authors as needed. While data extraction and analysis were duplicated (NU and MH/DS), the precision of the data was verified by another author (HP). We were aware of the potential impact of reflexivity while analyzing and interpreting the qualitative data. Nonetheless, the involvement of a multidisciplinary author group proficient in qualitative evidence synthesis helped ensure a balanced interpretation of the data.

### Interpretation in the Light of Published Literature

#### Effectiveness of Asynchronous Digital Health

Our study indicates that asynchronous digital health can effectively complement or replace other consultation approaches for asthma care in diverse settings. This extends the findings of a recent Cochrane review on digital interventions specifically for improving asthma treatment adherence [15]. All studies included in the meta-analysis demonstrated improved asthma control despite substantial heterogeneity. Our sensitivity analysis confirmed that the heterogeneity was likely attributable to 1 study, possibly because the reported values were derived from model estimates [74]. Another study, not included in the meta-analysis but considered in the sensitivity analysis, revealed a decline in asthma control [78]. This incongruity could be linked to the study's combined reporting of ACT and C-ACT measures despite the known differences in their performance [78].

Our study showed no significant difference in quality of life compared to usual care. In contrast, a scoping review focused on a different health condition using asynchronous digital health demonstrated improved self-efficacy and quality of life [82]. Similarly, we found no significant change in emergency department visits, consistent with a systematic review indicating that health care utilization, specifically physician visits, did not decrease significantly. However, this review did observe a decline in visits among back pain and asthma patients, although these differences were not statistically significant [83]. Our study indicated a significant reduction in hospitalization risk, consistent with other reviews on conditions like rheumatoid arthritis, diabetes, and skin diseases [82-84].

#### Views and Experiences of Patients and Health Care Professionals

In our review, asynchronous digital health was favored by most parents of children with asthma and some adult patients, offering convenience for asthma management alongside work and family commitments. They valued remote monitoring, guidance from health care professionals, and self-management support. A study involving individuals with diabetes highlighted the benefits of asynchronous communication that complemented clinic visits and boosted patients' sense of responsibility for managing their conditions [85]. Health care professionals in our study believed asynchronous digital health could enhance communication and improve compliance and treatment adherence. Although they acknowledged convenience, they noted that remote reviews might not always be clinically suitable. Our findings align with a Cochrane review indicating that while 2-way text-based communication strengthens patient-provider relations [86], face-to-face consultations remain vital for certain cases, such as patients with complex symptoms or new cases [87]. The potential for misunderstandings in written digital communication was also recognized by clinicians, particularly for diabetes and young individuals with long-term conditions [88,89]. Similar sentiments were echoed in another study, emphasizing that asynchronous communication is not suitable for everyone and everything [90]. Therefore, it is important to align priorities between patients and health care professionals to establish a therapeutic partnership in digital health [91]. In our study, health care professionals perceived barriers to implementation, including workflow issues, lack of EHR integration, high workload, and absence of financial reimbursement. These barriers and challenges are consistent with other qualitative studies [87,92].

#### Process of Care Outcomes

A systematic review reported that several publications, mostly on the use of telehealth in dermatology and some that assessed multiple medical specialties, reported a positive impact on process of care outcomes, including shorter wait times and less time to perform a consultation [84]. Most asynchronous digital health cases had a total turnaround time of less than 72 hours, which is similar to our review finding [93].

### Implications for Clinical Practice and Policy Making

This systematic review gives confidence that asynchronous digital health can be an effective adjunct to other modes of consultation for asthma care. This could be especially valuable for patients and health care professionals with existing familiarity with digital technologies. Some health care systems have introduced guidelines, such as requiring at least 1 in-person visit before an e-visit, to establish patient-clinician relationships [94]. Successful integration of asynchronous digital health into routine practice must be designed to benefit patients, health care professions and organizations. Efficient task allocation and organization of care are crucial implementation facilitators. User-friendly digital features are essential, including tailored functionalities for 2-way asynchronous communication within set time frames, enabling questionnaire completion, and uploading images and videos (eg, inhaler technique) as necessary. Thus, patients, health care professionals and organizations can harness the benefit of asynchronous digital health for asthma care.

### Future Research

Further research is necessary to understand the organizational context and track the process of arranging, deploying and sustaining asynchronous consultations for asthma care. Moreover, there is a need for research to establish the best practices for implementing asynchronous digital health, engaging patient and public involvement groups, preventing misuse, and assessing the suitability of individuals with asthma or their caregivers and health care professionals across various health care settings. Identifying a low-risk group of individuals who could be managed appropriately through asynchronous digital consultations is another critical aspect requiring investigation.

### Conclusions

Our review concludes with low confidence that asynchronous consultation supported by digital functionalities is effective and convenient and can be considered as an option for non-emergency asthma care, showing good acceptability among individuals with asthma and their caregivers. Thus, asynchronous digital consultations present an opportunity for those whose lifestyles or geographical locations impede synchronous consultations but require organization strategies to manage workload.

## Appendix

Multimedia Appendix 1 Supplementary materials.

Multimedia Appendix 2 PRISMA checklist.

## Abbreviations

ACQ: asthma control questionnaire
ACT: asthma control test
AQLQ: asthma quality of life questionnaire
C-ACT: childhood-ACT
EHR: electronic health record
GRADE: Grading of Recommendations, Assessment, Development, And Evaluations
GRADE-CERQual: GRADE-Confidence in the Evidence from Qualitative Reviews
MAQLQ: mini AQLQ
PAQLQ: pediatric AQLQ
PRISMA: Preferred Reporting Items for Systematic Reviews and Meta-Analyses
RCT: randomized control trial
RR: risk ratio
SMD: standardized mean difference

## Data Availability

All data generated or analyzed during this study are included in this published article and its supplementary information files.
