# Supplementary material for: Effectiveness and Acceptability of Asynchronous Digital Health in Asthma Care: Mixed Methods Systematic Review
Source: J Med Internet Res. 2024 Dec 3;26:e57708. doi: 10.2196/57708 (PMC11653041; doi:10.2196/57708)
Supplement: Multimedia Appendix 1 [file jmir_v26i1e57708_app1.pdf]

Multimedia appendix 1

Table of contents

Appendix S1: Database search strategy ... 2
Appendix S2: Inclusion, exclusion criteria and operational rules ... 4
Appendix S3: Initial coding strategy ... 5
Appendix S4: List of excluded studies ... 7
Appendix S5: Summary of included quantitative studies and quantitative data from mixed-methods studies ... 10
Appendix S6: Summary of qualitative studies and qualitative data from mixed-methods studies ... 15
Appendix S7: Sensitivity analysis ... 18
Appendix S8: Risk of bias for individual clinical outcomes ... 19
Appendix S9: Narrative synthesis of clinical outcomes ... 20
Appendix S10: Digital health functionalities ... 21
Appendix S11: Methodological quality of qualitative studies ... 22
Appendix S13: Certainty of evidence for clinical outcomes ... 24
Appendix S14: Confidence in qualitative evidence ... 25
References ... 27

Appendix S1: Database search strategy

MEDLINE

1 exp Asthma/
2 asthma$.ti,ab,kw.
3 exp Bronchial Spasm/
4 exp Bronchoconstriction/
5 exp Bronchial Hyperreactivity/
6 wheez$.ti,ab,kw.
7 1 or 2 or 3 or 4 or 5 or 6
8 exp Telemedicine/
9 exp Telenursing/
10 exp Electronic Mail/
11 exp Digital Technology/
12 exp Smartphone/
13 exp Mobile Applications/
14 (telehealth or teleconsult$ or telemanagement or telemonitor$ or telecare or telematics or telepharmacy or telehomecare or telesupport or ehealth or e-health or econsult$ or evisit$ or e-visit$ or mhealth or m-health or web or web-based or ict or digital questionnaire or portal).ti,ab,kw.
15 ((online or remote or distant or asynchronous) adj3 (review$ or monitor$ check-up or follow-up or care or consult$)).ti,ab,kw.
16 8 or 9 or 10 or 11 or 12 or 13 or 14 or 15
17 17 7 and 16 1674
18 limit 17 to yr="2001 -Current"

Embase

1 exp asthma/
2 asthma$.ti,ab,kw.
3 exp bronchospasm/
4 exp bronchoconstriction/
5 exp bronchus hyperreactivity/
6 wheez$.ti,ab,kw.
7 1 or 2 or 3 or 4 or 5 or 6
8 exp telemedicine/
9 exp telenursing/
10 exp teleconsultation/
11 exp e-mail/
12 exp digital technology/
13 exp smartphone/
14 exp mobile application/
15 (telehealth or telemanagement or telemonitor$ or telecare or telematics or telepharmacy or telehomecare or telesupport or ehealth or e-health or econsult$ or evisit$ or e-visit$ or mhealth or m-health or web or web-based or ict or digital questionnaire or portal).ti,ab,kw.
16 ((online or remote or distant or asynchronous) adj3 (review$ or monitor$ check-up or follow-up or care or consult$)).ti,ab,kw.
17 8 or 9 or 10 or 11 or 12 or 13 or 14 or 15 or 16
18 7 and 17
19 limit 18 to yr="2001 -Current"

PsycInfo

1 exp Asthma/
2 (asthma$ or bronchospasm or bronchoconstrict$ or "bronchial hyperreactivity" or wheez$).tw.
3 1 or 2
4 exp Telemedicine/
5 exp Teleconsultation/
6 exp Digital Technology/
7 exp Smartphones/
8 exp Mobile Applications/
9 (telehealth or telemanagement or telenurs$ or telemonitor$ or telecare or telematics or telepharmacy or telehomecare or telesupport or ehealth or e-health or econsult$ or evisit$ or e-visit$ or mhealth or m-health or web or web-based or email or e-mail or ict or digital questionnaire or portal).tw.

10 ((online or remote or distant or asynchronous) adj3 (review$ or monitor$ check-up or follow-up or care or consult$)).tw.
11 4 or 5 or 6 or 7 or 8 or 9 or 10
12 3 and 11
13 limit 12 to yr="2001 -Current"

Scopus
(TITLE-ABS-KEY (asthma* OR bronchospasm OR bronchoconstrict* OR "bronchial hyperreactivity" OR wheez*) AND TITLE-ABS-KEY (telemedicine OR telehealth OR teleconsult* OR telemanagement OR telemonitor* OR telecare OR telematics OR telepharmacy OR telehomecare OR telesupport OR ehealth OR e-health OR econsult* OR e-consult* OR evisit OR e-visit OR mhealth OR m-health OR web OR "web-based" OR email OR e-mail OR ict OR "digital questionnaire*" OR portal OR telenurs* OR "digital technology" OR "smart phone" OR smartphone OR "mobile applications" OR ((online OR remote OR distant OR asynchronous) W/3 (review* OR monitor* OR check?up OR follow?up OR care OR consult*)))) AND PUBYEAR > 2000 AND PUBYEAR > 2000

CINAHL
S1 TI asthma* OR bronchospasm OR bronchoconstrict* OR "bronchial hyperreactivity" OR wheez
S2 AB asthma* OR bronchospasm OR bronchoconstrict* OR "bronchial hyperreactivity" OR wheez*
S3 S1 OR S2
S4 TI telemedicine OR telehealth OR teleconsult* OR telemanagement OR telemonitor* OR telecare OR telematics OR telepharmacy OR telehomecare OR telesupport OR ehealth OR e-health OR econsult* OR e-consult* OR evisit OR e-visit OR mhealth OR m-health OR web OR "web-based" OR email OR e-mail OR ict OR "digital questionnaire*" OR portal OR telenurs* OR "digital technology" OR "smart phone" OR smartphone OR "mobile applications" OR ((online OR remote OR distant OR asynchronous) N3 (review* OR monitor* OR check?up OR follow?up OR care OR consult*))
S5 AB telemedicine OR telehealth OR teleconsult* OR telemanagement OR telemonitor* OR telecare OR telematics OR telepharmacy OR telehomecare OR telesupport OR ehealth OR e-health OR econsult* OR e-consult* OR evisit OR e-visit OR mhealth OR m-health OR web OR "web-based" OR email OR e-mail OR ict OR "digital questionnaire*" OR portal OR telenurs* OR "digital technology" OR "smart phone" OR smartphone OR "mobile applications" OR ((online OR remote OR distant OR asynchronous) N3 (review* OR monitor* OR check?up OR follow?up OR care OR consult*))
S6 S4 OR S5
S7 S3 AND S6
S8 S7 (Published Date: January 2001 – present)

Cochrane library
#1 (asthma* OR bronchospasm OR bronchoconstrict* OR "bronchial hyperreactivity" OR wheez*):ti,ab,kw
#2 (telemedicine OR telehealth OR teleconsult* OR telemanagement OR telemonitor* OR teleconsult* OR telecare OR telematics OR telepharmacy OR telehomecare OR telesupport OR ehealth OR e-health OR econsult* OR e-consult* OR evisit OR e-visit OR mhealth OR m-health OR web OR "web-based" OR email OR e-mail OR ict OR "digital questionnaire*" OR portal OR telenurs* OR "digital technology" OR "smart phone" OR smartphone OR "mobile applications" OR ((online OR remote OR distant OR asynchronous) NEAR/3 (review* OR monitor* OR check?up OR follow?up OR care OR consult*))):ti,ab,kw
#3 #1 AND #2
#4 #3 with Cochrane Library publication date Between Jan 2001 and present

Appendix S2: Inclusion, exclusion criteria and operational rules

	Inclusion criteria	Exclusion criteria	Operational rules
Population	• Children (and their caregivers) and adults with a primary diagnosis of asthma<br>• Comorbidity will not be an exclusion criterion as long as the focus of the intervention is asthma	• Studies that recruited participants with other long-term conditions, unless they report data for people with asthma.	..
Intervention	• Asynchronous digital consultation for asthma care<br>• Concomitant face-to-face or synchronous consultations will not be an exclusion criterion as long as a proportion of the care is provided by asynchronous digital interventions	• Exclusively synchronous or real-time consultations by any means such as face-to-face consultations, video-conferences, telephone calls etc.<br>• Acute asthma consultations	**Asynchronous digital consultation:** Patients or their caregivers and healthcare providers use digital tools to communicate with each other without engaging in immediate, real-time conversations (e.g., e-Consultations)<br>**Asthma care:** Communications between patients and their healthcare providers to review asthma status, excluding emergency situations
Comparison (Quantitative study)	• Either population receiving 'Usual care' OR<br>• receiving care exclusively by 'synchronous consultations'<br>OR<br>• no review consultation	..	**'Usual Care'**- is the standard face-to-face consultation received by an individual with asthma in the any healthcare system<br>**'Synchronous consultations'**- are the real-time or concurrent asthma care by any mode of consultation.
Outcomes (Quantitative study)	One or more of the following outcomes:<br>**Clinical outcomes:** Asthma control, Quality of life, Acute attacks<br>**Process outcomes:** Health service time (e.g., time to first response, duration of consultation), Patient time (e.g., time completing consultation tasks)	..	Clinical outcome measurement:<br>Priority was given to validated instruments for measuring asthma control (e.g., Asthma Control Test)[32] or quality of life (e.g., Mini–Asthma Quality of Life Questionnaire) [33], and acute attacks as defined by ATS/ERS Task force and measured by unscheduled healthcare visits/admissions requiring a steroid course[34].
Phenomena of interest (Qualitative study)	Studies that explored views and experiences of patients, and/or professional stakeholders on asynchronous digital consultation (with or without other modes of communication) for asthma care	Studies that did not include any views or opinions about asynchronous consultations	Data about the views and experiences of patients or their caregivers, and/or professional stakeholders on asynchronous digital health (either as an isolated intervention or as an adjunct to other forms of consultation) for asthma care.
Setting and context	Any countries and healthcare settings irrespective of economic status	..	..
Study designs	Quantitative, quantitative and mixed-methods studies	Case study, case report, editorials, letter to editor, commentary, reviews, expert opinion articles, and conference abstracts.	..
Language	• Quantitative studies: no language restriction<br>• Mixed-methods studies: no language restriction for quantitative component<br>• Qualitative studies: English only	Qualitative studies and qualitative component of mixed-methods studies published in languages other than English	..

Appendix S3: Initial coding strategy

**Andrews 2019** [52]	**Doyle 2019** [58]	**Haze 2013** [62]	**Hui 2021** [63]	**Nichols 2020** [66]	**Schneider 2016** [70]	**van der Meer 2007** [75]
• Preferred electronic communication, either text messages, application-based messages, or e-mails, others phone call<br>• Contact to come from someone at their child's physician's office<br>• Preferred for 2-way communication<br>• Several parents want a tool that could track the child's asthma information.<br>• Parents recommended incorporating allergen alerts, peak flow tracking, and daily medication reminders into the application<br>• Many parents were comfortable or preferred at least monthly risk communication<br>• Some participants preferred to be contacted by text message or smartphone application<br>• Some prefer a phone call.	• Liked the idea of using SMS to support asthma control, could be helpful reminder<br>• Most said they would be fine responding to these messages, but some said they would ignore the messages if they felt like their asthma was well controlled or if they were busy and did not have time to answer immediately.<br>• One adult said he would only respond if he was asked a simple yes or no question.<br>• Submitting an ACT via SMS every month: Almost all participants were willing to submit a monthly score, but two adults admitted that they might not actually respond to these requests<br>• Desire for tailored messages, both in terms of the length/frequency of the program and the content of the messages<br>• Preference to receive messages on Monday, to start the week, and then again on Thursday or Friday, to start the weekend.<br>• Both adolescent and young adult participants liked the idea of	Patient<br>• perception of positive change in the nurse-patient relationship<br>• more comfortable communicating with text messaging and believed that they were ''able to ask more questions''<br>• improved access was the reason for an improvement in the relationship with the RN<br>• Application allowed teenagers to initiate communication to RN care coordinators outside scheduled clinic hours, at the time that was most convenient for them<br>**HCPs**<br>• teenagers likely responded more honestly about their asthma control<br>• comments sent via text messaging more accurately reflected and addressed the control assessment<br>• seemed to respond with more detail through text messaging<br>• If the teenager was actively using the application, the RN care coordinator knew	Patient<br>• Online information was of interest, ideally personalised to clinical context and individual situation<br>Clinicians<br>• Professionals considered that reputable information such as inhaler technique videos, treatment information, why and how the medication should be taken were important for patient self-management<br>Digital action plan<br>• Most patients wanted a (digital) action plan to remind them what to do when they forgot the agreed actions when their conditions were getting worse<br>• Most clinicians suggested an action plan to remind about medication adjustment and agreed actions if patients' condition was getting worse<br>**Monitoring condition with feedback**<br>• Usefulness of remote monitoring<br>• Medication adherence logs<br>Regular clinical review	• Participants conveyed strong support for the reminder alerts in facilitating autonomy and in transitioning from parent-managed to shared responsibility<br>• Older adolescents expressed that they were able to take on a more active role and expanded autonomy through self-management of medication administration and symptom management. (Views on technology)<br>Views and experience<br>• Participants expressed a strong comfort level with the SAMS system following the initial demonstration, with several sharing similar feedback that it was quite easy to use.<br>• All participating children shared the sentiment that it was easy to use and highlighted the use of technology as a normative component of their daily lives.<br>• The features that contributed to the increased competence in managing asthmatic symptoms included educational content and the ability to tailor feedback following the video capture of the use of inhaler.<br>• For the video capture of the inhaler use technique, participants were prompted with a series of on-screen prescripted prompts to promote proper inhaler use techniques.<br>• Tailored feedback was provided by the respiratory therapist following a review of the video capture feature within the app.<br>• The tailored feedback included tips on proper inhalation techniques such as mouth placement on the inhaler, holding one's breath after inhalation, and shaking the inhaler before use.	• Current communication with adolescent patients is limited, given demanding schedules, the brevity of most clinic visits, and the protracted intervals between consecutive visits<br>• Mobile technology could improve communication by reaching adolescents directly<br>• Perceived text messaging and e-mails as efficient and preferred communication channels to connect with patients.<br>• A feasible, accessible, and acceptable outreach mode<br>• Can convey accurate and credible information	• Poorly-controlled asthma: Few were bothered by the face-to-face medical reviews, since they had learned to live with their symptoms and saw no need for regular consultations<br>• Most participants: not necessary to visit their physician if their asthma was under control<br>• Few mentioned that doctor visits were annoying.They preferred to visit their doctor only when symptoms were getting worse.<br>• Participants were enthusiastic about the internet-based review of lung function by sending lung function values and symptom scores to their physician via the internet or SMS, with the possibility of adding comments or questions<br>• Patients with both poorly and well-controlled asthma mentioned that e-mail communication and electronic consultation was useful.<br>• Almost everyone used the computer daily.<br>• Most participants felt no need to see their

• The remaining stated they are okay with any method of contact (text, application, e-mail, call).<br>• Most parents reported checking their e-mail "frequently," but this could mean once a week to several times a day.<br>• Most parents reported that they have had the same phone number for several years.<br>• A few parents reported using applications for communication with their children's teachers and rated that experience favourably and can see similarities to how an asthma application could be useful	receiving messages during times of day when they would be available to read the messages; either early in the morning before school/work, in the evening after school/ work, or a few participants also wanted to receive messages around lunchtime.<br>• Most participants like to receive messages between once a week and every day.<br>• Several participants said the messages could be better received if they were sent less frequently and could be less predictable if the message frequency is reduced over time<br>• Participants liked the idea of choosing the length of the program themselves based on their own needs.<br>**Message Content**<br>• preferred messages with cues to action to take their controller medication, visit their clinic, or refill their inhaler	the teenager was available for contact with current access to their cell phone.<br>• The RN care coordinator could then call the teenager to ask more questions, reverting to synchronous communication	• Most patients wanted reminders for the yearly review.<br>• Some preferred web-based or teleconsultation for regular reviews to save travel time<br>• Most professionals thought reminders would encourage attendance, and agreed remote consultations were convenient though not always clinically appropriate<br>**Communication with health care professionals**<br>• Most patients wanted flexibility to ask quick follow up questions, and a patient with hearing problems found WhatsApp useful<br>• Clinicians agreed with a flexible approach to reviews, including text services for quick follow up questions, though resources would be needed	• This was particularly true for one newly diagnosed child, as he initially ran out of medication early because of improper technique. (See Components, see van Gaalen 2013)<br>• Others describe the benefit of capturing inhaler use for technique refinement and parental ability to ensure proper medication administration adherence<br>• The participating children shared a sense of pride and accomplishment in self-managing their care, an essential factor in enhanced competence, which can facilitate increased engagement<br>• Feeling supported and cared for by others were strong attributes of relatedness throughout the intervention and, in general, participants appreciated concern over health and well-being conveyed by others while participating in the study.<br>• Others expressed appreciation in knowing that providers were actively involved and concerned with their child's well-being and care management<br>Benefits<br>• Convenience was another benefit shared by the participants.<br>• Parents articulated how they often struggle to manage work and family responsibilities and how finding time to have a conversation during the work day with a provider can be challenging<br>• Felt that SAMS facilitated communication in a convenient manner for all parties.<br>• Improved day-to-day care management and reduced unplanned provider visits, indicative of fewer asthmatic exacerbations for the children and the ability for parents to reduce time lost from work<br>• The use of technology to increase medication adherence and accuracy of medication administration was supported by children and parents.		physician or nurse in person for regular review<br>Benefits<br>• Majority thought IBSM: feasible, not time consuming, did not interfere daily activities, easy and fast to share monitoring scores<br>• All preferred to receive information through internet rather than leaflets or books

Appendix S4: List of excluded studies

S/N	Title	Author	Reason for exclusion
1	Investigating the attitude of patients with chronic diseases about using mobile health	Abbasi 2020	Not relevant intervention
2	The impact of using a mobile application to improve asthma patients' adherence to medication in Jordan	Al-Nawayseh 2021	Not relevant intervention
3	Opinions of healthcare providers on the design of a smartphone application for asthma treatment in the Kingdom of Saudi Arabia	Al-Otaibi 2020	Not relevant intervention
4	Multidisciplinary Consensus for the Monitoring and Control of Asthma Through Telemedicine. The COMETA Project	Sánchez 2021	Not relevant study design
5	Evaluation of internet use for health information by parents of asthmatic children attending pediatric clinics in Riyadh, Saudi Arabia	AlSaadi 2012	Not relevant intervention
6	Feasibility of Collecting Diary Data From Asthma Patients Through Mobile Phones and SMS (Short Message Service): Response Rate Analysis and Focus Group Evaluation From a Pilot Stud	Anhøj 2004	Not relevant intervention
7	Patient Portal Usage and Outcomes Among Adult Patients with Uncontrolled Asthma	Apter 2020	Not relevant intervention
8	Computer-Assisted School-Based Asthma Management: A Pilot Study	Arnold 2012	Not relevant intervention
9	Social Media, Text Messaging, and Email—Preferences of Asthma Patients between 12 and 40 Years Old	Baptist 2011	Not relevant outcome
10	Internet-Based Self-Management Support After High-Altitude Climate Treatment for Severe Asthma: Randomized Controlled Trial	Beerthuizen 2020	Not relevant intervention
11	Adults With Asthma Experience No Increase in Asthma-related Exacerbations When Digital Communication Technology Tools Are Employed to Offset Provider Workload: A Pragmatic Randomized Trial	Bender 2020	Not relevant intervention
12	WeChat Public Account Use Improves Clinical Control of Cough-Variant Asthma: A Randomized Controlled Trial	Cao 2018	Not relevant population
13	Transition to Virtual Asthma Care During the COVID-19 Pandemic: An Observational Study	Cvietusa 2022	Not relevant intervention
14	A consumer designed smartphone app for young people with asthma: pilot of engagement and acceptability	Davis 2019	Not relevant intervention
15	Home telemonitoring (forced expiratory volume in 1 s) in children with severe asthma does not reduce exacerbations	Deschildre 2011	Not relevant intervention
16	Using Mobile Health to Improve Asthma Self-Management in Early Adolescence: A Pilot Randomized Controlled Trial	Fedele 2021	Not relevant intervention
17	Improving Pediatric Asthma Control Among Minority Children Participating in Medicaid: Providing Practice Redesign Support to Deliver a Chronic Care Mode	Fifield 2010	Not relevant intervention
18	A Shared e-Decision Support Portal for Pediatric Asthma	Fiks 2014	Not relevant study design
19	Development and implementation of the home asthma telemonitoring (HAT) system to facilitate asthma self-care	Finkelstein 2001	Not relevant outcome
20	Improving knowledge and decreasing depressive symptoms in caregivers of children with asthma through the asthma academy: A randomized controlled trial	Foronda 2021	Not relevant intervention
21	Asthma self-management app for Indonesian asthmatics: A patient-centered design	Ghozali 2021	Not relevant study design
22	Effect of the School-Based Telemedicine Enhanced Asthma Management (SB-TEAM) Program on Asthma Morbidity: A Randomized Clinical Trial	Halterman 2018	Not relevant intervention
23	Telemedicine use for pediatric asthma care: a mixed methods study	Haynes 2021	Not relevant intervention
24	Patients' and Clinicians' Perceived Trust in Internet-of-Things Systems to Support Asthma Self-management: Qualitative Interview Study	Hui 2021	Not relevant phenomena of interest

25	Time to change the paradigm? A mixed method study of the preferred and potential features of an asthma self-management app	Hui 2020	Not relevant intervention
26	Use of Online Self-Management Diaries in Asthma and COPD: A Qualitative Study of Subjects' and Professionals' Perceptions and Behaviors	Kruijssen 2015	Not relevant phenomena of interest
27	Planning for Action: The Impact of an Asthma Action Plan Decision Support Tool Integrated into an Electronic Health Record (EHR) at a Large Health Care System	Kuhn 2015	Not relevant intervention
28	A Web-based mobile asthma management system	Lee 2005	Not relevant outcome
29	Asthma Management Using the Mobile Asthma Evaluation and Management System in China	Lin 2021	Not relevant intervention
30	Phase II trial of web-based tailored asthma management intervention in adolescents at clinics.	Lu 2019	Not relevant intervention
31	A randomized controlled trial of a mobile application-assisted nurse-led model used to improve treatment outcomes in children with asthma	Lv 2019	Not relevant intervention
32	School nurse perception of asthma care in schoolbased telehealth	MacGeorge 2021	Not relevant intervention
33	Caring for Children with Asthma through Teleconsultation: "ECHO-Pac, The Electronic Children's Hospital of the Pacific"	Malone 2004	Not relevant intervention
34	Going mobile with primary care: smartphone telemedicine for asthma management in young urban adults (TEAMS)	Mammen 2020	Not relevant intervention
35	Engagement in "My Child's Asthma", an interactive web-based pediatric asthma management intervention	Meischke 2011	Not relevant outcome
36	Assessing the Needs and Perspectives of Patients With Asthma and Chronic Obstructive Pulmonary Disease on Patient Web Portals: Focus Group Study	Metting 2018	Not relevant outcome
37	Exploring the perspectives of clinical professionals and support staff on implementing supported self-management for asthma in UK general practice: an IMP2ART qualitative study	Morrow 2017	Not relevant phenomena of interest
38	Telemonitoring in asthma control: a randomized controlled trial	Nemanic 2018	Not relevant intervention
39	Understanding the potential role of mobile phone-based monitoring on asthma self-management: qualitative study	Pinnock 2007	Not relevant phenomena of interest
40	The use of text messaging to improve asthma control: a pilot study using the mobile phone short messaging service (SMS)	Prabhakaran 2010	Not relevant intervention
41	Effectiveness of the eCARE programme: a short message service for asthma monitoring	Prabhakaran 2019	Not relevant intervention
42	Giving Asthma Support to Patients GASP): a novel online asthma education, monitoring, assessment and management tool	Ram 2014	Not relevant intervention
43	Adolescent Preferences and Design Recommendations for an Asthma Self-Management App: Mixed-Methods Study	Roberts 2018	Not relevant intervention
44	Adolescent, caregiver, and friend preferences for integrating social support and communication features into an asthma self-management app	Roberts 2016	Not relevant intervention
45	Adolescent feedback on predisposing, reinforcing and enabling features in asthma self-management apps	Roberts 2019	Not relevant intervention
46	Core Components for a Clinically Integrated mHealth App for Asthma Symptom Monitoring	Rudin 2017	Not relevant intervention
47	Clinical and cost effectiveness of mobile phone supported self-monitoring of asthma: multicentre randomised controlled trial	Ryan 2012	Not relevant intervention
48	I have most of my asthma under control and I know how my asthma acts: Users' perceptions of asthma self-management mobile app tailored for adolescents	Schneider 2020	Not relevant phenomena of interest
49	Online Support Intervention for Adolescents With Asthma and Allergies: Ingredients and Insights	Stewart 2011	Not relevant intervention
50	Understanding reasons for asthma outpatient (non)-attendance and exploring the role of telephone and e-consulting in facilitating access to care: exploratory qualitative study	Baar 2006	Not relevant phenomena of interest
51	The effectiveness of nurse-led telemonitoring of asthma: results of a randomized controlled trial	Willems 2007	Not relevant intervention
52	Process evaluation of a nurse-led telemonitoring programme for patients with asthma	Willems 2007	Not relevant intervention

53	Patient–Clinician Mobile Communication: Analyzing Text Messaging Between Adolescents with Asthma and Nurse Case Managers	Yoo 2013	Not relevant outcome
54	Telehealth to improve asthma control in pregnancy: A randomized controlled trial	Zairina 2016	Not relevant intervention
55	Asthma Management Using the Mobile Asthma Evaluation and Management System in China	Lin 2022	Not relevant intervention
56	Partnered Decision Support: Parental Perspectives of Completing a Pre-Visit Pediatric Asthma Questionnaire via the Patient Portal	Ross 2022	Not relevant phenomena of interest
57	The feasibility and impact of implementing a computer-guided consultation to target health inequality in Asthma	Chakrabarti 2023	Not relevant intervention
58	Impact of a mobile-app assisted self-management educational intervention on the scores of asthma control test (ACT) questionnaire among young asthmatic patients.	Ghozali 2023	Not relevant intervention

Appendix S5: Summary of included quantitative studies and quantitative data from mixed-methods studies

**Author year**<br>Country, setting<br>Design (duration): groups<br>RoB/quality	**Participant**<br>(Age, enrolled, attrition)	**Intervention**			**Clinical outcomes**<br>(Asthma control, QoL, ED visits, hospitalisations)	**Comments**
		**Asynchronous digital communication**	**Other digital functionalities**	**Non-digital support**		
**Ahmed 2016** [51]<br>Canada, two tertiary care hospitals<br>RCT (6m): DHI vs UC<br>RoB: High risk for QoL	Adults (18-69y) with asthma<br>Enrolled: 100 (DHI: 49, UC: 51)<br>Attrition: DHI: 17 (35%), UC: 3 (6%)	Web-portal mail with a nurse case-manager (response within 24 hours)	• View personal health information<br>• Asthma information<br>• Weekly data submission via web-portal with CDSS generated alerts	• Regular 'visits'<br>• Nurse-initiated telephone calls were an option in response to alerts	Asthma control (Poor control status) *: Between group OR (95% CI): 0.94 (0.33 to 2.71)<br>QoL (MAQLQ)*: Between group MC (95% CI): 0.22 (–0.34 to 0.78)<br>ED visits: no difference (reported narratively)<br>Hospitalisations: NR	No significant between group differences in all clinical outcomes. Greater attrition in the DHI group.
**Chan 2007** [55]<br>USA, Military health service, Oahu;<br>RCT (12m): DHI vs UC;<br>RoB: High risk for QoL, some concerns for ED visits and hospitalisation	Children (6-17y) with persistent asthma<br>Enrolled: 120 (DHI: 60, UC: 60);<br>Attrition<br>DHI: 13 (22%), UC: 5 (8%)	Web-based asthma communication and email with asthma case managers (24hours/day for emergency care)	• Three virtual visits: education<br>• Web-based symptom diary and quality of life survey<br>• Transfer of recorded peak flow and inhaler use videos for feedback	• Three FtF visits (UC had 6 FtF visits)<br>• Option of telephone calls for emergency situations	QoL (PAQLQ)*: MC (SD): DHI: 0.1 (1.0), UC: 0.1 (1.2)<br>ED visits (n): DHI: 4, UC: 2<br>Hospitalisations (n): DHI: 1, UC: 1<br>Asthma control: NR	No significant between group differences in all clinical outcomes. Greater attrition in the DHI group.
**Cingi 2015** [57]<br>Turkey, University respiratory clinics;<br>RCT (3m): DHI vs UC;<br>RoB: High risk for asthma control, ED visits	Adults (25-41y) with mild-to-severe persistent asthma<br>Enrolled: 136 (DHI: 68, UC: 68;<br>Attrition: DHI: 8 (12%), UC: 39 (57%)	Mobile phone app messaging:<br>• urgent messages<br>• information<br>• receive physician's review	• Monitor health status<br>• Medication reminders<br>• Motivational nudges	..	Compared to UC, DHI group were more likely to achieve asthma control (ACT>19) *: 49% vs 27% (*P*< 0.05)<br>QoL, ED visits, Hospitalisations: NR	Significant between group differences favouring DHI. Large attrition from UC group.
**Fiks 2015** [60]<br>USA, primary care practices<br>RCT (6 m):<br>DHI vs UC<br>RoB: Some concerns for asthma control; low for ED visits and hospitalisations	Children (6-12y) with persistent asthma and their parents/guardians<br>Enrolled: 60 (DHI: 30, UC: 30)<br>Attrition: DHI: 4 (13%), UC: 3 (10%)	Web-portal for communication:<br>• monthly asthma control survey<br>• CDSS supported response from clinician	• Asthma information<br>• Parental and children's goals<br>• Action plan	Telephone calls were an option for responding to poor control	Asthma control (PACT): Between group DID (Flares): -2.0; *P*=.02<br>QoL (ITGCA-SF): Between group DID (Functional): 4.3, *P* =.4<br>ED visits (n): DHI: 3, UC: 9<br>Hospitalisations: DHI: 0, UC: 2	Significant between group differences favouring DHI for asthma control, but no significant changes for QoL and acute attacks
**Gustafson 2012** [61]<br>USA: Five MCOs in urban and rural area<br>RCT (12m):<br>DHI vs UC<br>RoB: Some concerns for asthma control	Children (4–12y) with asthma and their parents/guardians<br>Enrolled: 301 (DHI: 148, UC: 153)<br>Attrition: DHI: 16 (11%), UC: 26 (17%)	• Web portal mail system (response within 48 hours)	• Asthma information<br>• Monitoring<br>• Peer support<br>• Motivation<br>• Adherence coach	Monthly telephone calls from asthma nurse case manager	Asthma control (ACQ)*:<br>Between group DID (95% CI): -0.31 (-0.56 to -0.06)<br>QoL, ED visits, Hospitalisations: NR	Significant between group differences favouring DHI for asthma control

**Kosse 2019** [65]<br>Netherlands: 66 community pharmacies<br>Cluster RCT (6m):<br>DHI vs UC<br>RoB: Some concerns for asthma control and QoL	Adolescents (12-18y) with asthma.<br>Enrolled: 253 (DHI: 103, UC: 150)<br>Attrition: DHI: 16 (16%), UC: 3 (2%)	Mobile phone app-based communication with pharmacists.	• Asthma information<br>• Peer support (chat)<br>• weekly control questions<br>• medication reminders<br>• bi-weekly medication adherence questions	Regula 'visits'	Asthma control (CARAT): Between group effect size (95% CI): +0.23 (−0.47; 0.93)<br>QoL (PAQLQ): Between group effect size (95% CI): +0.03 (−0.13; 0.20)<br>ED visits and Hospitalisations: NR<br>Self-reported medication adherence (MARS)*: Between group effect size (95% CI): +0.60 (−0.43; 1.63)	Participants well matched at baseline.<br>No significant between group differences for asthma control and QoL
**Ostojic 2005** [67]<br>Croatia: hospital clinic<br>Pilot RCT (4m)<br>DHI vs UC<br>RoB: High for hospitalisations	Young adults 24.6y (SD 6.5) with moderate/ persistent asthma<br>Enrolled: 16 (DHI: 8, UC: 8)<br>Attrition: 0	Mobile SMS:<br>• send daily peak flow data<br>• receive weekly instructions from an asthma specialist<br>• attack alerts	..	FtF consultations provided if peak flow rate <50% predicted	Hospitalisations (n): DHI: 2, UC: 7<br>Asthma control, QoL and ED visits: NR	Significant between group reduced risk for hospitalisation favouring DHI
**Rasmussen 2005** [68]<br>Denmark: Catchment area of a University Hospital<br>RCT (6m): DHI[IG] vs UC[GP] (vs SG not relevant to this review)<br>RoB: Some concerns for ED visits	Adults (18-45y) with persistent asthma<br>Enrolled: 300 (DHI[IG]: 100, UC[GP]: 100, SG: 100)<br>Attrition: DHI[IG]: 15 (15%), UC[GP]: 20 (20%), SG: 12 (12%),	Web-portal:<br>• fill in daily e-Diary<br>• receive email instructions from physician on treatment	• Web-based action plan<br>• CDSS to guide specialist advice on treatment	Telephone call option for communication	QoL (AQLQ)*: Between group OR (95% CI) 2.10 (1.02 to 4.31)<br>ED visits: DHI: 2, UC: 1<br>Unscheduled visits: DHI: 3.7%, UC: 1.3%<br>Asthma control: NR	Significant between group difference favouring DHI for QoL
**van den Wijngaart 2017** [72]<br>Netherlands: General hospitals and tertiary referral centres<br>RCT (16m):<br>DHI vs UC<br>RoB: Some concerns for asthma control, low for ED visits and hospitalisations	Children (6–16y) with asthma<br>Enrolled: 210 (DHI: 105, UC: 105)<br>Attrition: DHI: 4 (4%), 12 (11%)	• Web-portal mail with HCPs (response within 48 hours)<br>• Feedback on monthly questionaries'	• Daily symptoms questions, and monthly (C-)ACT<br>• Online action plan<br>• Peer support (chat forum)<br>• Asthma information	Two FtF visits (UC had 4 FtF visits)<br>HCP contact triggered by poor control	Between group difference<br>Asthma control<br>• C-ACT: Difference (95% CI): 1.17 (0.09–2.25), *P* =.03<br>• ACT: Difference (95% CI) 0.88 (−0.41–2.16), *P* =.18<br>ED visits: RR (95% CI): 1.50 (0.26–8.79), *P* =.67<br>Hospitalisations: RR (95% CI): 0.50 (0.05–5.43), *P* =.56<br>QoL: NR<br>Unscheduled visits: RR (95% CI): 1.35 (0.88–2.07), *P* = .24<br>SFDs*: Difference (95% CI): 1.23 (0.42–2.04), *P* =.003	Significant between group difference in asthma control among 6-11 years age group favouring DHI, but no significant differences were found for other clinical outcomes
**van der Meer 2009** [74]<br>Netherlands: 37 General practices and one academic outpatient department<br>RCT (12 months):<br>DHI vs UC<br>Downs and Black score: 22	Adults (18 - 50y) with asthma<br>Enrolled: 200 (DHI: 101, UC: 99)<br><br>Attrition: DHI: 10 (10), UC: 7 (7%)	Web-portal mail, mobile SMS with asthma nurse	• Weekly ACQ and AQLQ<br>• Daily symptoms and lung function<br>• Online education<br>• CDSS to guide HCPs advice on treatment	• Regular visits<br>• TCs were an option to communicate with asthma nurse	Between group difference (95% CI)<br>Asthma control (ACQ):<br>-0.47 (-0.64 to -0.30), *P* <0.001<br>QoL (AQLQ)*: 0.38 (0.20 to 0.56), *P* <0.001<br><br>ED visits, Hospitalisations: NR	Significant between group differences in clinical outcomes favouring DHI

**Apter 2019** [53]<br>USA, outpatient practices of an academic medical centre and a primary care practice<br>RCT (12m):<br>Supported DHI+^{CHW} vs DHI^{UC}<br>Downs and Black score: 24	Adults (≥18y) with moderate/severe asthma<br>Enrolled: 301 (DHI+^{CHW} 151, DHI^{UC} 150)<br>Attrition: 28 (DHI+^{CHW} 16 (11%), DHI^{UC} 12 (8%)	Web portal communication with HCPs.<br>Both groups had access, but only the DHI+^{CHW} group were supported in use of the web-portal	• Access general asthma information<br>• View EHR and review test results<br>• Order refills<br>• Make appointments	• 4 home visits over first 6m by CHWs to train in use of portal + care co-ordination + action plan.<br>• Quarterly telephone calls	Supported DHI+^{CHW} vs DHI^{UC}<br>DID (95% CI)<br>Asthma control (ACQ)*: -0.21 (-0.56 to 0.15)<br>QoL (AQLQ): +0.31 (-0.13 to 0.79)<br>ED visits: -0.60 (-2.21 to 0.97)<br>Hospitalisations: -0.53 (-1.08 to -0.024)	Usage of portal similar between groups.<br>Clinical outcomes improved in both groups, with no significant between group differences
**Voorend-van Bergen 2015** [78]<br>Netherlands: General hospitals and tertiary referral centres<br>RCT (12 months):<br>DHI vs UC (vs FG not relevant to this review)<br>RoB: Some concerns for asthma control and QoL	Children (4–18y) with atopic asthma<br>Enrolled: 272 (DHI: 91, UC: 89, FG: 92)<br>Attrition: DHI: 1 (1%), UC: 2 (1%), FG: 1 (1%)	Web-based communication via email (response within 72 hours)	• Monthly C-ACT/ACT<br>• Web-based symptoms diary<br>• CDSS to guide physician advice on treatment	• 3 FtF visits (4-monthly)	Between group difference (95% CI)<br>Asthma control [(ACT or C-ACT (combined)): 0.09 (−0.92 to 1.11), *P* =.86<br>QoL (PAQLQ): −0.05 (−0.34 to 0.23), *P* =.72<br>ED visits and Hospitalisations: NR<br>SFDs*: −6.60 (−15.5 to 2.3), *P* =.15	8 poor adherers excluded after 4-week run-in.<br>ACT and C-ACT scores combined despite not being fully comparable [32,35].<br>No separate data available.<br>Minimal description of the DHI.
**Wiecha 2015** [79]<br>USA, community health centres and other practices in Boston area<br>Pilot RCT (6m):<br>DHI vs UC<br>Downs and Black score: 21	Children (9-17y) with persistent asthma<br><br>Enrolled: 58 (IG: 37, UC: 21)<br><br>Attrition: IG: 9 (24%, UC: 7 (33%)	Web-portal discussion board with physician and asthma nurse on treatment advice	• Asthma education<br>• Peak flow and symptoms monitoring<br>• Alerts for red zone peak flow values	Two home visits: Baseline survey, dose counter attachment to controller medication, and web portal training for participants	ED visits: no difference (reported narratively)<br>Asthma control, QoL, Hospitalisation: NR<br>Primary controller compliance*:<br>DHI: MC +29.8, p=0.01, UC: MC −5.0, *P* =.81	No significant between groups differences in ED visits. Controller medication adherence improved significantly in DHI group.
**Xu 2010** [80]<br>Australia, Royal Children's Hospital Brisbane, and Caboolture, Gold Coast, and Ipswich hospitals in Queensland<br>Pilot RCT (6 months):<br>DHI^{NSG} vs UC (vs DHI^{IVR} not relevant to this review)<br>Downs and Black score: 20	Children and young people (3-16y) with asthma<br><br>Enrolled: 121 (DHI^{NSG}: 41, UC: 41, DHI^{IVR}: 39)<br>Attrition: DHI^{NSG}: NR, UC: 1(2%), DHI^{IVR}: NR)	Communication with specialist nurse every 2 weeks via email: asthma symptoms, medication use, management plan and asthma education.	..	Telephone calls were an option to communicate with the asthma nurse	DHI^{NSG} vs UC<br>*ED visits: OR (95% CI) 1.71 (0.50, 5.81), *P* =.39<br>*Hospitalisations: OR (95% CI) 0.87 (0.20, 3.85), *P* =.85<br>QoL (PAQLQ): Between group MC (95% CI) 0.41 (−0.03 to 0.84)<br>Asthma control: NR	Effects of the intervention with asynchronous digital functionalities were not clearly outlined.
**Acharya 2018** [50]<br>USA, HIMSS Stage 6 regional trauma centres and associated clinical practices<br>Quantitative evaluation survey survey using 5 point Likert scale (12 months)<br>Downs and Black score: 6	Adults (18–44y) with persistent asthma, n=68<br>HCPs (gastroenterologists, lung specialists, emergency care, and/<br>or primary care providers), n=36<br>IT professionals, n=12	Communication with physician via mobile app-based text messaging	• Asthma information<br>• Peak flow, symptoms, medications<br>• Action plan<br>• Inhaler counter<br>• Call 911 in emergency	..	Clinical outcomes: NR<br><br>50% of the patients, and 83% of the HCPs agreed that Pro-care reduces the number of office visits	Findings were mostly presented qualitatively.

**Arimova 2021** [54]<br>Russia, Central Clinical Hospital of the Russian Academy of Sciences<br>Observational comparative study (6 months): DHI vs UC<br>Downs and Black score: 8	Patients with asthma aged 4-17 years<br><br>Enrolled: 68 (DHI: 41, UC: 27)<br><br>Attrition: NR	Communication with physician via mobile Chat bot	• Daily peak flow<br>• Monthly C-ACT/ACT<br>• Data submission reminder	..	Asthma control (C-ACT/ACT): asthma was under control in 70% of patients in DHI group,<br>QoL, ED visits, Hospitalisations: NR<br>Compliance to self-control tools*:<br>51% patients filled in peak flow data in DHI, none in the UC;<br>46% of patients filled C-ACT/ACT in DHI, none in the UC (*P* =.0001);<br>Patients under 12, who performed peak flow with their parents, did it 7 times more frequently than teenagers (who performed peak flow alone)	Originally in Russian, this paper was translated to English using Google Translate, and the extracted data were subsequently verified by a native Russian speaker.<br><br>Greater adherence to self-control tools in DHI.
**Chan 2003** [56]<br>USA, settings: NR<br>Pre-post study (6 months)<br>Downs and Black score: 10	Children (6-17y) with mild to moderate persistent asthma<br>Enrolled: 10<br>Attrition: 0	Web-based asthma communication and email with asthma case manager (pharmacist)	• Daily asthma symptom diary<br>• Twice weekly transfer of recorded peak flow and inhaler use videos for feedback	• 5 FtF clinic visit (0, 2, 6, 12, and 24 weeks)<br>• Telephone calls were an option to communicate with case manager	Asthma control: NR<br>QoL (PAQLQ):<br>Mean (SD): Pre: 6.5 ± 0.5; post: 6.6 ± 0.4<br>ED visits: 0<br>Hospitalisations: 0	No change in QoL
**Kosse 2019 [**64]<br>Process evaluation of a Cluster RCT[67]	Adolescents (12-18y) with asthma<br>Included 87 patients (55% females) who completed the intervention; 86 actively used the downloaded app	Mobile phone app-based communication with pharmacists.	• Asthma information<br>• Peer support (chat)<br>• weekly control questions<br>• medication reminders<br>• Bi-weekly medication adherence questions	Regula 'visits'	Clinical outcomes reported in Kosse 2019 [65]<br>Process outcomes:<br>• Females used the app more often than males (*P* =.01)<br>• 13% of the adolescents used all functionalities of the app<br>• Total app usage had no impact on medication adherence, while pharmacist chat significantly improved adherence (*P* =.03)	Pharmacist chat had a significant positive impact on medication adherence.
**Rasulnia 2018** [69]<br>USA, Duke University Asthma, Allergy, and Airways Centre<br>Pre-post study (3 months)<br>Downs and Black score: 17	Patients (21 – 78y) with asthma<br>Enrolled: 51<br>Attrition: 11 (22%)	Communication with health advisor via mobile text message and email	..	• Printed asthma information booklet<br>• Telephone calls were an option to engage with health advisor	Asthma control (ACT):<br>MC (SD): 1.68 (4.0), *P* =.011<br>QoL (PROMIS-G10SF):<br>MC (SD): 1.55 (2.4), *P* =.0100<br>*ED visits or hospital:<br>MC(SD): 0.07 (0.9), *P* =1.000	Asthma control and QoL improved.<br>No difference in acute attacks requiring ED visits or hospitalisation
**van der Kamp 2021** [73]<br>Netherlands, Paediatric department of Medisch Spectrum Twente, Enschede	Children (4-18y) moderate to severe asthma<br>Enrolled: 35<br>Attrition: 5 (14%)	Communication with HCPs (nurse practitioners, technical physician, and paediatric pulmonologist) via a web-based app chat	• Share photos, videos, and sound recordings<br>• Share monitoring data<br>• Emergency button	..	Post-pre intervention:<br>• Asthma control (C-ACT): Relative difference (%): +9, *P* =.40<br>• QoL (EQ-5D): Relative difference (%): –2, *P* =.50<br>Compared to prior six months before the study began:	No significant change in asthma control and QoL.<br>ED visits and hospitalisations reduced substantially

Quasi-experimental single arm pre-post study (6 months)<br>Downs and Black score: 13					• ED visits: Difference (relative difference in %): –17 (–81)<br>• Hospitalisations: Difference (relative difference in %): –11 (–85)	
**van Gaalen 2013** [76]<br>Netherlands, 37 General practices and one academic outpatient department<br>Long-term follow-up of an RCT (van der Meer 2009): DHI vs UC<br>Downs and Black score: 19	Adults (18 - 50y) with asthma<br><br>Enrolled: 107 (IG: 47, UC: 60)<br>Invited: 200 (IG: 101, UC: 99)<br>Attrition at 30th months FU: DHI 54 (53) %, UC: 39 (39%)	Up to 12 months<br>Web-portal mail, mobile SMS with asthma nurse<br><br>No further intervention was provided	Up to 12 months<br>• Weekly ACQ and AQLQ<br>• Daily symptoms and lung function<br>• Online education<br>• CDSS to guide HCPs advice on treatment	Up to 12 months<br>• Regular visits<br>• Telephone calls were an option to communicate with asthma nurse	Follow-up at 30th months compared to baseline measurements<br>Between group difference (95%CI)<br>Asthma control (ACQ): -0.33 (-0.61 to -0.05)<br>QoL (AQLQ): 0.29 (0.01-0.57), *P* =.03<br>ED visits, Hospitalisations: NR	Between group significant improvement in asthma control and QoL favouring DHI
**Fiks 2016** [59]<br>USA, Primary care practices from two practice-based research networks<br>Mixed-methods implementation study, quantitative survey<br>MMAT: Some concerns	Parents or guardians of children (6-12y) with asthma<br>Out of 9133 eligible patients, 237 (2.59%) completed the portal asthma control survey at least once	Communication with HCPs via web-portal	• Asthma information<br>• Monthly asthma control survey<br>• Action plan<br>• CDSS to guide HCPs and parents on treatment advice	• Telephone calls were an option to communicate with HCPs	Asthma control, QoL, ED visits, Hospitalisations: NR<br>Difference between study year and previous year, N (%) of children (95% CI)<br>• Medication change: +11 [+14% (2 to 27)]<br>• Primary care visit: +12 [+16% (3 to 28)]	Portal users with uncontrolled asthma had significantly more medication changes and primary care asthma visits

*Authors reported primary outcome

Abbreviations: DHI: digital health intervention, UC: usual care, RoB: risk of bias, MMAT: mixed methods appraisal tool, CDSS: clinical decision support system, Ftf: face-to-face, ACT: asthma control test, C-ACT: childhood-ACT, PACT: paediatric asthma control tool, ACQ: asthma control questionnaire, CARAT: control of allergic rhinitis and asthma test, QoL: quality of life, AQLQ: asthma quality of life questionnaire, PAQLQ: paediatric asthma quality of life questionnaire, MAQLQ: mini asthma quality of life questionnaire, ITGCAF: integrated therapeutics group child asthma short form, PROMIS-G10SF: patient reported outcomes measurement information system global-10 short form, EQ-5D: Euroqol-5 dimensions, ED: emergency department, OR: odd ratio, MC: mean change, DID: difference in difference, RR: risk ratio, SD: standard deviation, CI: confidence interval, NR: not reported, MARS: medication adherence report scale, DHI^{IG}: internet group, UC^{GP}: general practitioner group, SG: specialist group, HCP: healthcare professional, SFD: symptom free day, DHI^{CHW}: DHI+ community health worker, EHR: electronic health record, FG: FeNO group, SMS: short message service, IVR: interactive voice response, NSG: nurse support group, HIMSS: healthcare information and management systems society, IT: information technology.

Appendix S6: Summary of qualitative studies and qualitative data from mixed-methods studies

**Author year**<br>Country, setting<br>Design, method<br>Methodological quality	**Participant characteristics and number (n)**	**Study aim**	**Main findings**	**Phenomena of interest (review authors)**	**Comments**
**Andrews 2019** [52]<br>USA, Tertiary-care university-affiliated paediatric medical centre<br>Qualitative, interviews<br>CASP: Some concerns	Parents of children (2-17y) with asthma, n=20	Insight into mobile technology usage among parents of children with asthma and their preferences for an asthma risk communication and adherence promotion intervention	Parents welcomed digital assistance for asthma management, preferring two-way communication with clinically trained providers via text messages or phone calls. Their daily use of smartphones ensured high electronic accessibility	Views on mobile technology-based asynchronous communication for asthma care	Most parents preferred electronic communication, either texts, app-based messages, or emails, and desired two-way interaction
**Doyle 2019** [58]<br>USA, Community and school-based clinics<br>Qualitative, individual and group interviews<br>CASP: Some concerns	Patients (13-40y) persistent asthma<br>• Individual interviews, n=28<br>• Five group interviews, n=15	Acceptance of a short messaging service intervention to support asthma management	Participants were receptive toward the SMS programme and supported the use of tailored and interactive messages. They preferred directive educational messages and cues to action, while general messages reminding them of their asthma diagnosis were viewed less favourably	Views on a text messaging programme	Most participants preferred individualised, interactive messages and were willing to submit their monthly ACT scores via SMS
**Nichols 2020** [66]<br>USA, Children's hospitals (South Carolina)<br>Qualitative, dyadic key informant interviews<br>CASP: Some concerns	Dyads: parents with children and adolescents (8-17y) with high-risk asthma<br><br>19 dyadic interviews, n=38	Perceptions of youths with high-risk asthma and their caregivers on the use of a smartphone app, Smartphone Asthma Management System, in the prevention and treatment of asthma symptoms, possible use of the app to improve self-management of asthma outside traditional clinical settings, and the impact of asthma on everyday life to identify potential needs for future intervention development	Both children and parents preferred to use technology to facilitate medication administration and disease management. Although parents still want the ability to personally interact with clinicians on an as-needed basis, children and parents articulated an inclination toward the use of technology to manage routine care and monitoring	Views and experience of smartphone app-based asynchronous communication to improve self-management	Participants perceived that the smartphone asthma management system facilitated communication in a convenient manner and improved medication adherence
**Schneider 2016** [70]<br>USA, urban academic medical centre (Florida)<br>Qualitative, individual, and dyadic interviews, focus group<br>CASP: Some concerns	Resident physicians (27–32y)<br>• Individual interviews, n=14<br>• Two dyadic interviews, n=4<br>Staff physician (aged mid-30s to mid-40s)<br>• One focus group, n=4<br>• Individual interviews, n=3	Physicians' receptivity to using mobile technology as a strategy in patient care for adolescents with asthma	While staff physicians expressed reservations, in contrast to residents, about expanding the use of mobile devices in asthma care, they acknowledged the potential benefits of implementing mobile technology to improve communication and enhance adolescents' responsibility for their asthma status. However, both groups raised concerns about financial reimbursement and data security.	Views on mobile technology-based asynchronous communication in patient care for adolescents with asthma	Resident physicians showed more readiness for incorporating mobile technologies into patient care than staff physicians, and perceived text messaging and emails as efficient and preferred communication channels to connect with patients

	• One dyadic interview, n=2				
**van den Wijngaart 2018** [71]<br>Netherlands, general and tertiary hospitals, both in urban and rural<br>Qualitative survey<br>CASP: Some concerns	HCPs<br>• Paediatric pulmonologist, n=14<br>• Paediatrician, n=19<br>• Nurse practitioner=7<br>• Paediatric respiratory nurse, n=11<br>Parents of children (mean age: 10.1y, SD 2.5) with asthma, n=66	Barriers and facilitators experienced by Dutch health care professionals and patients (or their parents) when implementing eHealth in routine paediatric asthma care	Utilised Grol and Wensing framework to identify barriers and facilitators. Major barriers included lack of interoperability, insufficient financial reimbursement, increased workload for professionals, and shifts in the patient-professional relationship. Key facilitators were training and support, positive professional attitudes toward eHealth, patient benefits, and the ability to personalise care and streamline tasks using the web-portal.	Views and experience in implementing a web-portal with asynchronous digital functionalities for routine asthma care	Major barriers: lack of financial reimbursement, lack of integration with EHR, high workload<br>Major facilitators: positive attitude of professionals, possibility to tailor care to individual patients
**van Gaalen 2016** [77]<br>Netherlands, Primary care within South Holland, urban and rural settings<br>Qualitative, interviews and focus groups<br>CASP: High quality	Patients (20-51y) with asthma<br>• Four FGDs, n=20<br>• Interviews, n=2<br>HCPs<br>• Four FGDs with GPs, n=16<br>• Two FGDs with practice nurses, n=8<br>• Interviews with GPs, n=5<br>• Interviews with practice nurse, n=5	Barriers among patients, GPs and practice nurses to implement internet-based self-management for asthma in primary care	Utilised Grol and Wensing framework to identify barriers. Patients cited a lack of partnership and perceived limited benefit, GPs noted low urgency and routine challenges, and practice nurses highlighted low structured care and lack of support as barriers to PatientCoach (web-portal). Common barriers included usability issues, financial reimbursements, and patient characteristics.	Views on barriers related to the implementation of a web-portal with asynchronous digital functionalities in asthma care within general practices	Major barriers: lack of a patient–professional partnership, a low sense of urgency towards asthma care in current work routines, low level of structured asthma care, and lack of financial arrangements
**Fiks 2016** [59]<br>USA, Primary care urban, suburban and rural settings<br>Mixed-methods implementation study, interviews and FGDs<br>MMAT: Some concerns	Parents or guardians of children (6-12y) with asthma; and clinicians<br>• Interviews with parents, n=22<br>• 10 FGDs with clinicians, n=46	Feasibility of using a patient portal for paediatric asthma in primary care, its impact on management,<br>and barriers and facilitators of implementation success	Utilised the conceptual model of factors affecting the implementation of health innovations. Parents, especially those with children with uncontrolled asthma, were motivated to continue using the portal because it facilitated a better understanding and tracking of asthma control. Clinicians underscored the importance of coordinated practice workflows, including practice responsiveness to portal surveys to implementation success.	Views and experience regarding barriers and facilitators in implementing a web portal with asynchronous digital functionalities for asthma care in primary care practices	Qualitative findings highlighted the importance of practice organisation, family responsiveness, and innovation characteristics to portal adoption
**Hui 2021** [63]<br>UK, primary and secondary care<br>Exploratory sequential mixed-methods study, think-aloud qualitative interviews<br>MMAT: High quality	Patients (16y and above) with asthma, interviews, n=12<br><br>HCPs, interviews, n=12 (GP=2, Asthma nurse=2, Consultant chest physician=1, Asthma	Patients' and clinicians' preferences for a future internet-of-things system and explore their visions of its potential to support holistic self-management	Used PRISMS taxonomy for findings. Both patients and clinicians preferred automated monitoring with real-time feedback in an IoT system that could support a wide range of self-management tasks. Patients wanted a system to log their asthma control status automatically, provide real-time advice to help them learn about their asthma, identify and avoid triggers, and adjust their treatment. Clinicians desired automated objective patient data for consultations. Clinicians appreciated the potential reduction in face-	Views on the design and utility of the internet of things with asynchronous digital functionalities as a strategy for supporting self-management	Patients felt secure with remote clinical communication through text or email. Clinicians sought automated, objective patient data and appreciated the prospect of fewer in-person consultations.

	paediatrician=3, Pharmacist=4)		to-face consultations, seeing it as a time and resource-saving measure.		
**Haze 2013** [62]<br>USA, primary care<br>Mixed- methods study, semi-structured interviews<br>MMAT: Low quality	Patients (13-18y) with persistent asthma, interviews, n=20<br>Registered nurse care coordinators, interviews, n=2	Feasibility and efficacy of using a smartphone app to communicate between a patient with asthma and their nurse care coordinator.	Teenagers with asthma felt they could ask more questions and access quicker responses using the smartphone app. Nurse care coordinators noted enhanced ability to contact teens and improved accuracy of assessment data. Smartphone technology and text messaging enhanced the nurse-patient relationship.	Views and experience in using a smartphone app with asynchronous digital functionalities for asthma care	Both patients and nurses recognised that the smartphone app improved access to care, and text messaging enhanced communication
**van der Meer 2007** [75]<br>Netherlands, general practices (in and around Leiden)<br>Mixed- methods study, FGDs<br>MMAT: Some concerns	Patients (12-17y) with asthma, eight FGDs, n=35	Barriers and benefits perceived by adolescents with well-controlled and poorly controlled asthma to current and internet-based asthma management	Used theory of planned behaviour to report findings. Most participants found internet-based monitoring and reporting feasible, mentioning it as non-time-consuming and non-interfering to daily activities. Sending lung function values and symptom scores via the internet or SMS was described as easy and fast. Patients in the well-controlled group enjoyed the measurements but did not perceive benefits from daily electronic monitoring and feedback due to the absence of current symptoms.	Views on internet-based self- management programme with asynchronous digital functionalities for asthma care	Most adolescents preferred seeking medical help only when symptoms worsened, and appreciated internet-based reviews, email communication, and electronic consultations

Abbreviations: CASP: critical appraisal skills programme, MMAT: mixed methods appraisal tool, ACT: asthma control test, SMS: short message service, EHR: electronic health record, HCP: healthcare professional, FGD: focus group discussion, GP: general practitioner, PRISMS: practical reviews in self-management support

Appendix S7: Sensitivity analysis

Asthma control (analysis 1)

Study or Subgroup	Intervention Mean	Intervention SD	Intervention Total	Usual care Mean	Usual care SD	Usual care Total	Weight	Std. Mean Difference IV, Random, 95% CI
Fiks 2015 (PACT)	2.8	4.97	26	0.6	7.49	27	10.1%	0.34 [−0.20, 0.88]
Gustafson 2012 (ACQ)	0.42	1.11	132	0.11	1.13	127	49.5%	0.28 [0.03, 0.52]
van den Wijngaart 2017 (ACT)	1.2	4.63	46	1	5.34	42	16.9%	0.04 [−0.38, 0.46]
van den Wijngaart 2017 (C-ACT)	1.7	4.26	59	1.2	5.59	63	23.5%	0.10 [−0.26, 0.45]
**Total (95% CI)**			**263**			**259**	**100.0%**	**0.20 [0.03, 0.37]**

Heterogeneity: $Tau^2 = 0.00$; $Chi^2 = 1.50$, df = 3 (P = 0.68); $I^2 = 0\%$
Test for overall effect: Z = 2.29 (P = 0.02)

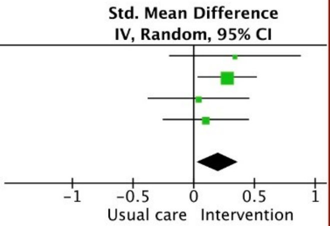

Asthma control (analysis 2)

Study or Subgroup	Intervention Mean	Intervention SD	Intervention Total	Usual care Mean	Usual care SD	Usual care Total	Weight	Std. Mean Difference IV, Random, 95% CI
Fiks 2015 (PACT)	2.8	4.97	26	0.6	7.49	27	7.5%	0.34 [−0.20, 0.88]
Gustafson 2012 (ACQ)	0.42	1.11	132	0.11	1.13	127	36.9%	0.28 [0.03, 0.52]
van den Wijngaart 2017 (ACT)	1.2	4.63	46	1	5.34	42	12.6%	0.04 [−0.38, 0.46]
van den Wijngaart 2017 (C-ACT)	1.7	4.26	59	1.2	5.59	63	17.5%	0.10 [−0.26, 0.45]
Voorend-van Bergen 2015 (ACT or C-ACT)	0.1	5.24	90	0.3	5.11	87	25.5%	−0.04 [−0.33, 0.26]
**Total (95% CI)**			**353**			**346**	**100.0%**	**0.14 [−0.01, 0.29]**

Heterogeneity: $Tau^2 = 0.00$; $Chi^2 = 3.39$, df = 4 (P = 0.50); $I^2 = 0\%$
Test for overall effect: Z = 1.85 (P = 0.06)

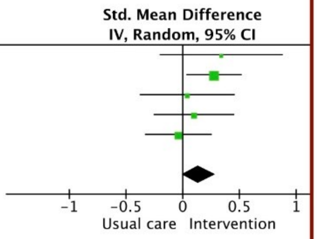

Emergency department visits

Study or Subgroup	Intervention Events	Intervention Total	Usual care Events	Usual care Total	Weight	Risk Ratio M-H, Random, 95% CI
Chan 2007	4	47	2	55	25.2%	2.34 [0.45, 12.21]
Fiks 2015	3	26	9	27	37.2%	0.35 [0.11, 1.14]
Rasmussen 2005	2	85	1	80	14.6%	1.88 [0.17, 20.36]
van den Wijngaart 2017	3	105	2	105	23.0%	1.50 [0.26, 8.79]
**Total (95% CI)**		**263**		**267**	**100.0%**	**1.01 [0.37, 2.77]**
Total events	12		14			

Heterogeneity: $Tau^2 = 0.35$; $Chi^2 = 4.45$, df = 3 (P = 0.22); $I^2 = 33\%$
Test for overall effect: Z = 0.01 (P = 0.99)

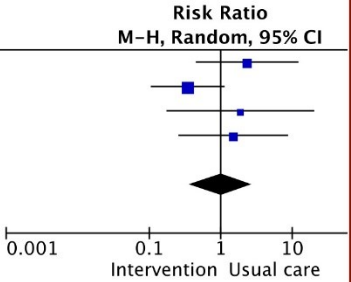

Hospitalisations

Study or Subgroup	Intervention Events	Intervention Total	Usual care Events	Usual care Total	Weight	Risk Ratio M-H, Random, 95% CI
Chan 2007	1	47	1	55	31.6%	1.17 [0.08, 18.20]
Fiks 2015	0	26	2	27	26.6%	0.21 [0.01, 4.12]
van den Wijngaart 2017	1	105	2	105	41.8%	0.50 [0.05, 5.43]
**Total (95% CI)**		**178**		**187**	**100.0%**	**0.52 [0.11, 2.42]**
Total events	2		5			

Heterogeneity: $Tau^2 = 0.00$; $Chi^2 = 0.71$, df = 2 (P = 0.70); $I^2 = 0\%$
Test for overall effect: Z = 0.84 (P = 0.40)

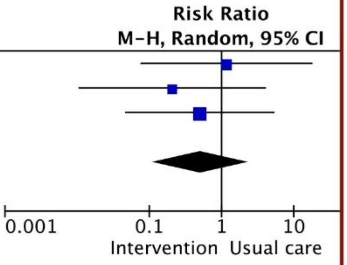

Appendix S8: Risk of bias for individual clinical outcomes

Parallel trial	Outcome	D1	D2	D3	D4	D5	Overall
Cingi 2015	Asthma control	+	+	-	!	!	-
Fiks 2015	Asthma control	+	+	+	!	+	!
Gustafson 2012	Asthma control	+	+	+	!	+	!
van den Wijngaart 2017	Asthma control	+	+	+	!	+	!
van den Wijngaart 2017	Asthma control	+	+	+	!	+	!
van der Meer 2009	Asthma control	+	+	+	!	+	!
Voorend-van Bergen 2015	Asthma control	+	+	+	!	!	!
Ahmed 2016	Quality of life	+	+	!	!	+	-
Chan 2007	Quality of life	!	+	!	!	+	-
van der Meer 2009	Quality of life	+	+	+	!	+	!
Voorend-van Bergen 2015	Quality of life	+	+	+	!	+	!
Chan 2007	Emergency department visits	!	+	+	+	+	!
Cingi 2015	Emergency department visits	+	+	-	+	+	-
Fiks 2015	Emergency department visits	+	+	+	+	+	+
Rasmussen 2005	Emergency department visits	+	+	!	+	+	!
van den Wijngaart 2017	Emergency department visits	+	+	+	+	+	+
Chan 2007	Hospitalisations	!	+	+	+	+	!
Fiks 2015	Hospitalisations	+	+	+	+	+	+
Ostojic 2005	Hospitalisations	!	+	+	!	+	-
van den Wijngaart 2017	Hospitalisations	+	+	+	+	+	+

Cluster trial	Outcome	D1a	D1b	D2	D3	D4	D5	Overall
Kosse 2019	Asthma control	+	!	+	+	!	+	!
Kosse 2019	Quality of life	+	!	+	+	!	+	!

D1(a): Randomisation process, D1b: Timing of identification or recruitment of participants, D2: Deviations from the intended interventions, D3: Missing outcome data, D4: Measurement of the outcome, D5: Selection of the reported result

+ Low risk
! Some concerns
- High risk

Appendix S9: Narrative synthesis of clinical outcomes

Among the quantitative studies that were excluded from the meta-analysis due heterogeneity in study design or data reporting revealed following results:
Asthma control: Two studies reported between group differences (intervention versus usual care) [51,76]. Of these studies, one study reported a statistically significant improvement (ACQ: RR -0.33; 95% CI -0.61 to -0.05) [76] in asthma control favouring intervention whereas the other found no significant change (unclear data reporting) [51]. Three studies reported within group changes before and after the intervention [53,69,73]. Of these studies, two found significant improvement in asthma control ((ACQ: MD -0.27; 95% CI -0.52 to -0.01) [53] and (ACT: MD±SD 1.68±4.0; P=0.011) [69]) and whereas the other found no significant change (C-ACT: relative difference +9%; P=0.40) [73].

Quality of life: Three studies reported between group differences (intervention versus usual care) [60,68,76]. Of these studies, two reported a statistically significant improvement ((AQLQ: RR 0.29; 95% CI 0.01 to 0.57; P=0.03) [76], (AQLQ: OR 2.10; 95% CI 1.02 to 4.31); P=.04) [68]) in quality-of-life favouring intervention whereas the other found no significant change (used the Integrated Therapeutics Group Child Asthma Short Form and reported three domains separately) [60]. Two studies reported within group changes before and after the intervention. Of these studies, one found significant improvement in quality of life (AQLQ: MD 0.41; 95% CI 0.10 to 0.71) [53] whereas the other found no significant change (EuroQol-5D: relative difference -2%; P=0.50) [73].

Emergency department visit: Three studies reported within group changes before and after the intervention [53, 69, 79]. Of these studies, two found no significant change ((MD±SD: 0.07±0.9; P=1.00) [69], and reported narratively [79]) whereas the other reported a reduction in emergency department visit (rate per year: change -2.69; 95% CI -3.67 to -1.88) [53]. One study narratively reported that there was no significant between group changes in emergency department visit [51].

Appendix S10: Digital health functionalities

Of the included quantitative studies (n=20) and one mixed-methods study that reported digital health functionalities, 15 (71%) used web-based portals [51,53,55,56,59-61,68,72-74,76,78-80], four (19%) used mobile applications [50,54,57,65], and two (10%) used mobile short message services (SMS) based interventions [67,69]. Only three (14%) studies were linked with existing electronic health records [51,59,60]. The frequently used digital functionalities were monitoring diary (n=14) [50,51,54-56,59-61,67,68,73,74,76,79], asthma control assessment (n=10) [54,57,59,60,65,72-74,76,78], education materials (n=9) [51,59-61,65,73,74,76,79], action plan (n=7) [50,51,59,60,68,72,79], automated feedback (n=7) [51,59,60,68,72,74,76], medication reminder (n=4) [54,57,65,67], videos of PEF and inhaler technique transfer (n=2) [55,56], communication with healthcare professionals (HCPs): online chat (n=12) [50,53,54,57,59,60,65,72-74,76,79], email (n=12) [51,55,56,59-61,68,69,74,76,79,80], telephone (n=7) [55,56,68,69,74,76,80], SMS (n=4) [67,69,74,76]. Consultation task completion times varied widely across the studies, both for patients and healthcare professionals. Patients' engagement ranged from an average of 7 minutes/login [51] to 15 minutes/week [73] and 26 minutes/week [67]. Physicians spent on an average 2 minutes/week/patient [67], while nurses invested 1-2 hours per week [74]. Asthma severity, younger age, and higher parental education correlated with frequent portal use [59]. Females used the app more often (median 20.5 vs 11; P=.01) and for longer durations (median 5 vs 6 months; P=.03) than males [64].

Appendix S11: Methodological quality of qualitative studies

Study ID	Was there a clear statement of the aims of the research?	Is a qualitative methodology appropriate?	Was the research design appropriate to address the aims of the research?	Was the recruitment strategy appropriate to the aims of the research?	Was the data collected in a way that addressed the research issue?	Has the relationship between researcher and participants been adequately considered?	Have ethical issues been taken into consideration?	Was the data analysis sufficiently rigorous?	Is there a clear statement of findings?	Overall assessment
Andrews 2019 [52]	Yes	Yes	Can't tell (Study design was not justified)	Can't tell (reported recruitment process, but no description about sampling strategy, declined participants)	Yes	Yes	Yes	Yes	Yes	Some concerns
Doyle 2019 [58]	Yes	Yes	Can't tell (Study design was not justified)	Can't tell (reported recruitment process, but no description about why they recruited them)	Yes	No (Reflexivity was not reported)	Yes	Yes	Yes	Some concerns
Nichols 2020 [66]	Yes	Yes	Can't tell (Study design was not justified	Yes	Yes	No (Interviewer's role was not described)	Yes	Yes	Yes	Some concerns
Schneider 2016 [70]	Yes	Yes	Can't tell (Study design was not justified	Yes	Yes	No (Interviewer's role was not described)	Yes	Yes	Yes	Some concerns
van den Wijngaart 2018 [71]	Yes	Yes	Can't tell (Study design was not justified	Yes	Can't tell (Data collection method was not justified)	No (Reflexivity was not reported)	Yes	Yes	Can't tell (Supporting quotes were not provided, reported following Grol and Wensing framework)	Some concerns
van Gaalen 2016 [77]	Yes	Yes	Yes	Yes	Yes	Yes	Yes	Yes	Yes	High quality

Appendix S12: Methodological quality of mixed-methods study

Study ID	Is there an adequate rationale for using a mixed methods design to address the research question?	Are the different components of the study effectively integrated to answer the research question?	Are the outputs of the integration of qualitative and quantitative components adequately interpreted?	Are divergences and inconsistencies between quantitative and qualitative results adequately addressed?	Do the different components of the study adhere to the quality criteria of each tradition of the methods involved?
Haze 2013 [62]	Yes (Described rationale of using mixed-methods, but briefly)	No (No attempt to integrate the quantitative and qualitative findings)	No	No	No
Fiks 2016 [59]	Yes (Described in the introduction section)	No (No explicit attempt to integrate the quantitative and qualitative findings)	Yes (The conceptual model of factors affecting the implementation of health innovations was efficiently used to interpret the qualitative and quantitative findings)	No	Yes (Quantitative component was described well. The conduction of a few focus groups over phone raises some concerns)
Hui 2021 [63]	Yes (Described in the introduction section)	Yes (Explicitly integrated the quantitative and qualitative component)	Yes (Triangulated the survey findings with interview themes and interpreted adequately)	Yes (Reported and explained)	Yes (Both components were well described)
van der Meer 2007 [75]	Yes (Described rationale of using mixed-methods)	Yes (Explicitly integrated the qualitative and quantitative)	Yes (Interpreted adequately)	No	Yes (Both components were well described)

Appendix S13: Certainty of evidence for clinical outcomes

Outcomes	Participants (studies)	Risk of bias	Inconsistency	Indirectness	Imprecision	Publication bias	Overall certainty of evidence
Asthma control	1045 (6 RCTs) [57,60,61,65,72,74]	very serious[a]	not serious	not serious	serious[b]	none	⨁◯◯◯ Very low
Quality of life	788 (5 RCTs) [51,55,65,74,78]	serious[c]	not serious	not serious	serious[b]	none	⨁⨁◯◯ Low
Emergency department visit	619 (5 RCTs) [55,57,60,68,72]	serious[d]	not serious	not serious	not serious	none	⨁⨁⨁◯ Moderate
Hospitalisation	381 (4 RCTs) [55,60,67,72]	serious[e]	not serious	not serious	serious[f]	none	⨁⨁◯◯ Low

Explanations

a. Two studies were deemed to have a high risk of bias: one due to missing outcome data and the other due to selective reporting. The 'outcome measurement' domain was judged to have some concerns in all studies because of using self-reported outcome measurement tools.
b. Risk of imprecision due to the use of standardised mean difference to assess the effect.
c. The 'outcome measurement' domain was judged to have some concerns in all studies because of using self-reported outcome measurement tools. Two studies raised some concerns about the randomisation process.
d. One study was deemed to have a high risk of bias due to missing outcome data, while another study raised some concerns. Two studies raised some concerns about the randomisation process.
e. Three studies raised some concerns about the randomisation process. One study was at low risk of bias for all domains.
f. One study included only a few participants.

Appendix S14: Confidence in qualitative evidence

Summary review findings	Studies contributing to findings	GRADE-CERQual assessment of confidence	Explanation of GRADE-CERQual assessment
**Acceptability of routine asthma reviews**			
Most adolescents perceived that in-person routine reviews were not necessary, particularly when their asthma was under control. Children including adolescents with asthma, their parents and some adult patients preferred remote reviews using digital health. Most healthcare professionals agreed remote reviews were convenient though not always clinically appropriate.	Hui 2021 [63]<br>van der Meer 2007 [75]<br>Nichols 2020 [66]	Moderate confidence	Moderate methodological limitations in all studies, with high coherence and adequacy and relevance in all studies. Represented 3 countries, primary and secondary care settings
**Advantages and disadvantages of asynchronous digital health**			
**Advantages**<br>Most parents of children with asthma found asynchronous digital health to be a convenient method for reviewing and managing their child's asthma while managing work and family responsibilities. They expressed a sense of reassurance in being able to remotely monitor their child's condition, receive guidance from healthcare professionals, and support self-management. Healthcare professionals thought that asynchronous digital health could enhance communication, and promote patient's compliance and treatment adherence.	Andrews 2019 [52]<br>Nichols 2020 [66]<br>Schneider 2016 [70]<br>van der Meer 2007 [75]	Moderate confidence	Moderate methodological limitations in all studies, with high coherence, adequacy and relevance in three studies. One study had moderate concerns about coherence and adequacy. Represented two countries, primary and secondary care settings.
**Disadvantages**<br>Healthcare professionals perceived that asynchronous digital health was not suitable for patients with poor perception of their asthma symptoms and for emergency cases. They also expressed concerns that the reduced face-to-face contact associated with this approach could negatively affect clinical decision-making. Some parents and children with asthma found frequent monitoring data sharing to be burdensome.	Nichols 2020 [66]<br>Schneider 2016 [70]<br>van den Wijngaart 2018 [71]	Low confidence	Serious methodological limitation in one study, moderate in two studies. One study had moderate concerns about coherence and adequacy. Relevance was moderate in all studies. Represented secondary care settings from two countries.
**Implementation barriers and facilitators**			
**Implementation barriers**<br>The major barriers to implementing asynchronous digital health, as perceived by most healthcare professionals, include poorly coordinated workflow, lack of integration with electronic health records, high workload, and the absence of financial reimbursement. Some healthcare professionals also mentioned that a lack of internet access and the unavailability of electronic devices or the lack of skills to use them by some patients acted as barriers.	Fiks 2016 [59]<br>van Gaalen 2016 [77]<br>van den Wijngaart 2018 [71]	High confidence	Methodological limitations - no or minor: 1, moderate: 1, serious: 1 with high coherence, adequacy and relevance in all studies. Represented two countries, primary and secondary care settings.

**Implementation facilitators**<br>Both healthcare professionals and parents of children with asthma recognised that accessible two-way communication, prompt responsiveness from practices and families, as well as a positive attitude and commitment towards adopting asynchronous digital health were facilitators. Healthcare professionals also emphasised the importance of efficient task allocation among the practice staff in facilitating successful implementation.	Andrews 2019 [52]<br>Fiks 2016 [59]<br>van den Wijngaart 2018 [71]	Moderate confidence	Methodological limitations - moderate: 1, serious: 1, with high coherence, adequacy and relevance in all studies. Represented two countries, primary and secondary care settings
**Preferred digital functionalities**			
Most patients wanted flexibility to ask quick questions, receive tailored information about asthma, log and visualise the trend of their peak flows, symptom scores, and medication usage. Healthcare professionals acknowledged the importance of a flexible approach to patient reviews but expressed concerns about available resources	Andrews 2019 [52]<br>Hui 2021 [63]<br>Nichols 2020 [66]<br>van den Wijngaart 2018 [71]	Moderate confidence	Methodological limitations: moderate: 3, serious: 1 (5172), with high coherence, adequacy and relevance in all studies. Represented three countries, primary and secondary care settings

References

(Numbered references correspond to citations within the manuscript)

32. Nathan RA, Sorkness CA, Kosinski M, Schatz M, Li JT, Marcus P, et al. Development of the asthma control test: a survey for assessing asthma control. J Allergy Clin Immunol. 2004 Jan;113(1):59-65. PMID: 14713908. doi: 10.1016/j.jaci.2003.09.008.
33. Juniper EF, Guyatt GH, Cox FM, Ferrie PJ, King DR. Development and validation of the Mini Asthma Quality of Life Questionnaire. Eur Respir J. 1999 Jul;14(1):32-8. PMID: 10489826. doi: 10.1034/j.1399-3003.1999.14a08.x.
34. Holguin F, Cardet JC, Chung KF, Diver S, Ferreira DS, Fitzpatrick A, et al. Management of severe asthma: a European Respiratory Society/American Thoracic Society guideline. Eur Respir J. 2020 Jan;55(1). PMID: 31558662. doi: 10.1183/13993003.00588-2019.
35. Liu AH, Zeiger R, Sorkness C, Mahr T, Ostrom N, Burgess S, et al. Development and cross-sectional validation of the Childhood Asthma Control Test. J Allergy Clin Immunol. 2007;119(4):817-25.
50. Acharya S, Sarraf R. A novel preventative solution for effective asthma management: a practical evaluation. Netw Model Anal Health Inform Bioinform. 2017;6(1):15. doi: http://dx.doi.org/10.1007/s13721-017-0156-7.
51. Ahmed S, Ernst P, Bartlett SJ, Valois M-F, Zaihra T, Pare G, et al. The Effectiveness of Web-Based Asthma Self-Management System, My Asthma Portal (MAP): A Pilot Randomized Controlled Trial. J Med Internet Res. 2016;18(12):e313.
52. Andrews AL, Nitchie HL, Harvey JB. Parent Preferences for Methods and Content of Mobile Technology-Based Asthma Medication Adherence Intervention. Hosp Pediatr. 2019;9(3):209-15. doi: https://dx.doi.org/10.1542/hpeds.2018-0122.
53. Apter AJ, Localio AR, Morales KH, Han X, Perez L, Mullen AN, et al. Home visits for uncontrolled asthma among low-income adults with patient portal access. J Allergy Clin Immunol. 2019;144(3):846-53. doi: https://dx.doi.org/10.1016/j.jaci.2019.05.030.
54. Arimova PS, Namazova-Baranova LS, Levina JG, Kalugina VG, Vishneva EA, Kharitonova EY. Mobile technologies in achieving and maintaining asthma control in children: First results of MedQuizBot Chat Bot. Pediatr Pharmacol. 2021;18(3):214-20. doi: http://dx.doi.org/10.15690/pf.v18i3.2279.
55. Chan DS, Callahan CW, Hatch-Pigott VB, Lawless A, Proffitt HL, Manning NE, et al. Internet-based home monitoring and education of children with asthma is comparable to ideal office-based care: results of a 1-year asthma in-home monitoring trial. Pediatrics. 2007;119(3):569-78.
56. Chan DS, Callahan CW, Sheets SJ, Moreno CN, Malone FJ. An Internet-based store-and-forward video home telehealth system for improving asthma outcomes in children. Am J Health Syst Pharm. 2003;60(19):1976-81.
57. Cingi C, Yorgancioglu A, Cingi CC, Oguzulgen K, Muluk NB, Ulusoy S, et al. The "physician on call patient engagement trial" (POPET): measuring the impact of a mobile patient engagement application on health outcomes and quality of life in allergic rhinitis and asthma patients. Int Forum Allergy Rhinol. 2015;5(6):487-97. doi: https://dx.doi.org/10.1002/alr.21468.
58. Doyle R, Albright K, Hurley LP, Chavez C, Stowell M, Dircksen S, et al. Patient perspectives on a text messaging program to support asthma management: A qualitative study. Health Promot Pract. 2019;20(4):585-92. doi: https://dx.doi.org/10.1177/1524839918770209.
59. Fiks AG, DuRivage N, Mayne SL, Finch S, Ross ME, Giacomini K, et al. Adoption of a Portal for the Primary Care Management of Pediatric Asthma: A Mixed-Methods Implementation Study. J Med Internet Res. 2016;18(6):e172. doi: https://dx.doi.org/10.2196/jmir.5610.
60. Fiks AG, Mayne SL, Karavite DJ, Suh A, O'Hara R, Localio AR, et al. Parent-reported outcomes of a shared decision-making portal in asthma: a practice-based RCT. Pediatrics. 2015;135(4):e965-73. doi: https://dx.doi.org/10.1542/peds.2014-3167.
61. Gustafson D, Wise M, Bhattacharya A, Pulvermacher A, Shanovich K, Phillips B, et al. The effects of combining Web-based eHealth with telephone nurse case management for pediatric asthma control: a randomized controlled trial. J Med Internet Res. 2012;14(4):e101. doi: https://dx.doi.org/10.2196/jmir.1964.
62. Haze KA, Lynaugh J. Building patient relationships: a smartphone application supporting communication between teenagers with asthma and the RN care coordinator. Comput Inform Nurs. 2013;31(6):266-71. doi: https://dx.doi.org/10.1097/NXN.0b013e318295e5ba.
63. Hui CY, McKinstry B, Fulton O, Buchner M, Pinnock H. Patients' and Clinicians' Visions of a Future Internet-of-Things System to Support Asthma Self-Management: Mixed Methods Study. J Med Internet Res. 2021;23(4):e22432. doi: https://dx.doi.org/10.2196/22432.
64. Kosse RC, Bouvy ML, Belitser SV, de Vries TW, van der Wa PS, Koster ES. Effective engagement of adolescent asthma patients with mobile health-supporting medication adherence. JMIR Mhealth Uhealth. 2019;7(3):e12411. doi: 10.2196/12411.

65. Kosse RC, Bouvy ML, de Vries TW, Koster ES. Effect of a mHealth intervention on adherence in adolescents with asthma: A randomized controlled trial. Respir Med. 2019;149:45-51. doi: https://dx.doi.org/10.1016/j.rmed.2019.02.009.
66. Nichols M, Miller S, Treiber F, Ruggiero K, Dawley E, Teufel R. Patient and parent perspectives on improving pediatric asthma self-management through a mobile health intervention: pilot study. JMIR Form Res. 2020;4(7):e15295. doi: 10.2196/15295.
67. Ostojic V, Cvoriscec B, Ostojic SB, Reznikoff D, Stipic-Markovic A, Tudjman Z. Improving asthma control through telemedicine: a study of short-message service. Telemed J E Health. 2005;11(1):28-35.
68. Rasmussen LM, Phanareth K, Nolte H, Backer V. Internet-based monitoring of asthma: a long-term, randomized clinical study of 300 asthmatic subjects. J Allergy Clin Immunol. 2005;115(6):1137-42.
69. Rasulnia M, Burton BS, Ginter RP, Wang TY, Pleasants RA, Green CL, et al. Assessing the impact of a remote digital coaching engagement program on patient-reported outcomes in asthma. J Asthma. 2018;55(7):795-800. doi: https://dx.doi.org/10.1080/02770903.2017.1362430.
70. Schneider T, Panzera AD, Martinasek M, McDermott R, Couluris M, Lindenberger J, et al. Physicians' perceptions of mobile technology for enhancing asthma care for youth. J Child Health Care. 2016;20(2):153-63. doi: https://dx.doi.org/10.1177/1367493514556555.
71. van den Wijngaart LS, Geense WW, Boehmer AL, Brouwer ML, Hugen CA, van Ewijk BE, et al. Barriers and Facilitators When Implementing Web-Based Disease Monitoring and Management as a Substitution for Regular Outpatient Care in Pediatric Asthma: Qualitative Survey Study. J Med Internet Res. 2018;20(10):e284. doi: https://dx.doi.org/10.2196/jmir.9245.
72. Van Den Wijngaart LS, Roukema J, Boehmer ALM, Brouwer ML, Hugen CAC, Niers LEM, et al. A virtual asthma clinic for children: fewer routine outpatient visits, same asthma control. Eur Respir J. 2017;50(4):1700471. doi: 10.1183/13993003.00471-2017.
73. van der Kamp M, Hartgerink PR, Driessen J, Thio B, Hermens H, Tabak M. Feasibility, efficacy, and efficiency of ehealth-supported pediatric asthma care: Six-month quasi-experimental single-arm pretest-posttest study. JMIR Form Res. 2021;5(7):e24634. doi: 10.2196/24634.
74. van der Meer V, Bakker MJ, van den Hout WB, Rabe KF, Sterk PJ, Kievit J, et al. Internet-based self-management plus education compared with usual care in asthma: a randomized trial. Ann Intern Med. 2009;151(2):110-20. doi: 10.7326/0003-4819-151-2-200907210-00008.
75. Van Der Meer V, Van Stel HF, Detmar SB, Otten W, Sterk PJ, Sont JK. Internet-based self-management offers an opportunity to achieve better asthma control in adolescent. Chest. 2007;132(1):112-9. doi: http://dx.doi.org/10.1378/chest.06-2787.
76. van Gaalen JL, Beerthuizen T, van der Meer V, van Reisen P, Redelijkheid GW, Snoeck-Stroband JB, et al. Long-term outcomes of internet-based self-management support in adults with asthma: randomized controlled trial. J Med Internet Res. 2013;15(9):e188. doi: http://dx.doi.org/10.2196/jmir.2640.
77. van Gaalen JL, van Bodegom-Vos L, Bakker MJ, Snoeck-Stroband JB, Sont JK. Internet-based self-management support for adults with asthma: a qualitative study among patients, general practitioners and practice nurses on barriers to implementation. BMJ Open. 2016;6(8):e010809. doi: https://dx.doi.org/10.1136/bmjopen-2015-010809.
78. Voorend-van Bergen S, Vaessen-Verberne AA, Brackel HJ, Landstra AM, van den Berg NJ, Hop WC, et al. Monitoring strategies in children with asthma: a randomised controlled trial. Thorax. 2015;70(6):543-50. doi: https://dx.doi.org/10.1136/thoraxjnl-2014-206161.
79. Wiecha JM, Adams WG, Rybin D, Rizzodepaoli M, Keller J, Clay JM. Evaluation of a web-based asthma self-management system: a randomised controlled pilot trial. BMC Pulm Med. 2015;15:17. doi: https://dx.doi.org/10.1186/s12890-015-0007-1.
80. Xu C, Jackson M, Scuffham PA, Wootton R, Simpson P, Whitty J, et al. A randomized controlled trial of an interactive voice response telephone system and specialist nurse support for childhood asthma management. J Asthma. 2010;47(7):768-73. doi: https://dx.doi.org/10.3109/02770903.2010.493966.
